# Glycosylation of immunoglobulin G is regulated by a large network of genes pleiotropic with inflammatory diseases

**DOI:** 10.1126/sciadv.aax0301

**Published:** 2020-02-19

**Authors:** Lucija Klarić, Yakov A. Tsepilov, Chloe M. Stanton, Massimo Mangino, Timo Tõnis Sikka, Tõnu Esko, Eugene Pakhomov, Perttu Salo, Joris Deelen, Stuart J. McGurnaghan, Toma Keser, Frano Vučković, Ivo Ugrina, Jasminka Krištić, Ivan Gudelj, Jerko Štambuk, Rosina Plomp, Maja Pučić-Baković, Tamara Pavić, Marija Vilaj, Irena Trbojević-Akmačić, Camilla Drake, Paula Dobrinić, Jelena Mlinarec, Barbara Jelušić, Anne Richmond, Maria Timofeeva, Alexander K. Grishchenko, Julia Dmitrieva, Mairead L. Bermingham, Sodbo Zh. Sharapov, Susan M. Farrington, Evropi Theodoratou, Hae-Won Uh, Marian Beekman, Eline P. Slagboom, Edouard Louis, Michel Georges, Manfred Wuhrer, Helen M. Colhoun, Malcolm G. Dunlop, Markus Perola, Krista Fischer, Ozren Polasek, Harry Campbell, Igor Rudan, James F. Wilson, Vlatka Zoldoš, Veronique Vitart, Tim Spector, Yurii S. Aulchenko, Gordan Lauc, Caroline Hayward

**Affiliations:** 1 Genos Glycoscience Research Laboratory, Zagreb, Croatia.; 2 MRC Human Genetics Unit, MRC Institute of Genetics and Molecular Medicine, University of Edinburgh, Edinburgh, UK.; 3 Institute of Cytology and Genetics, Siberian Branch of the Russian Academy of Science, 630090 Novosibirsk, Russia.; 4 Novosibirsk State University, 630090 Novosibirsk, Russia.; 5 Department of Twin Research and Genetic Epidemiology, King’s College London, London, UK.; 6 NIHR Biomedical Research Centre at Guy’s and St Thomas’ Foundation Trust, London, UK.; 7 Estonian Genome Center, University of Tartu, Tartu, Estonia.; 8 Institute of Molecular and Cell Biology, University of Tartu, Tartu, Estonia.; 9 Broad Institute of the Massachusetts Institute of Technology and Harvard University, Cambridge, MA, USA.; 10 Division of Endocrinology, Boston Children’s Hospital, Cambridge, MA, USA.; 11 Genomics and Biomarkers Unit, Department of Health, National Institute for Health and Welfare (THL), Helsinki, Finland.; 12 Molecular Epidemiology, Department of Biomedical Data Sciences, Leiden University Medical Centre, Leiden, Netherlands.; 13 Max Planck Institute for Biology of Ageing, Cologne, Germany.; 14 Faculty of Pharmacy and Biochemistry, University of Zagreb, Zagreb, Croatia.; 15 University of Split, Faculty of Science, Split, Croatia.; 16 Leiden University Medical Centre, Leiden, Netherlands.; 17 Division of Molecular Biology, Faculty of Science, University of Zagreb, Zagreb, Croatia.; 18 Colon Cancer Genetics Group, Cancer Research UK Edinburgh Centre and Medical Research Council Human Genetics Unit, Medical Research Council Institute of Genetics and Molecular Medicine, University of Edinburgh, Edinburgh, UK.; 19 Unit of Animal Genomics, WELBIO, GIGA-R and Faculty of Veterinary Medicine, University of Liège, Liège, Belgium.; 20 Centre for Global Health Research, Usher Institute of Population Health Sciences and Informatics, University of Edinburgh, Edinburgh, UK.; 21 Edinburgh Cancer Research Centre, Institute of Genetics and Molecular Medicine, University of Edinburgh, Edinburgh, UK.; 22 Department of Biostatistics and Research Support, University Medical Center Utrecht, Utrecht, Netherlands.; 23 CHU-Liège and Unit of Gastroenterology, GIGA-R and Faculty of Medicine, University of Liège, Liège, Belgium.; 24 Department of Public Health, NHS Fife, Kirkcaldy, UK.; 25 Department of Public Health, Faculty of Medicine, University of Split, Split, Croatia.; 26 Gen-info, Zagreb, Croatia.; 27 Psychiatric Hospital Sveti Ivan, Zagreb, Croatia.; 28 PolyOmica, Het Vlaggeschip 61, 5237 PA 's-Hertogenbosch, Netherlands.; 29 Kurchatov Genomics Center, Institute of Cytology & Genetics, Novosibirsk, Russia.; 30 Generation Scotland, Centre for Genomic and Experimental Medicine, Institute of Genetics and Molecular Medicine, University of Edinburgh, Edinburgh, UK.

## Abstract

Effector functions of immunoglobulin G (IgG) are regulated by the composition of a glycan moiety, thus affecting activity of the immune system. Aberrant glycosylation of IgG has been observed in many diseases, but little is understood about the underlying mechanisms. We performed a genome-wide association study of IgG N-glycosylation (*N* = 8090) and, using a data-driven network approach, suggested how associated loci form a functional network. We confirmed in vitro that knockdown of *IKZF1* decreases the expression of fucosyltransferase FUT8, resulting in increased levels of fucosylated glycans, and suggest that RUNX1 and RUNX3, together with SMARCB1, regulate expression of glycosyltransferase MGAT3. We also show that variants affecting the expression of genes involved in the regulation of glycoenzymes colocalize with variants affecting risk for inflammatory diseases. This study provides new evidence that variation in key transcription factors coupled with regulatory variation in glycogenes modifies IgG glycosylation and has influence on inflammatory diseases.

## Teaser

Variation in key transcription factors and glycogenes modifies IgG glycosylation and has an influence on inflammatory diseases.

## INTRODUCTION

Glycosylation, a series of reactions that creates complex carbohydrate structures (glycans) attached to a polypeptide backbone, is among the most common and complex posttranslational protein modifications. Highly regulated attachment of different glycans to the same glycosylation site (alternative glycosylation) can greatly contribute to variability in glycoprotein structure and influence function in a way that is analogous to changes in protein sequence (*1*). Immunoglobulin G (IgG) is a simple glycoprotein with only one conserved N-glycosylation site per heavy chain and glycans that have no more than two antennae, but even this simple glycosylation has the capacity to generate hundreds of different glycoforms of IgG. It is well established that alternative glycosylation of IgG can act as a molecular switch between different immune response outcomes and thus significantly affect the function of the immune system (*2*). Aberrant protein glycosylation has been observed in many physiological states, from aging- and age-related diseases to autoimmune diseases and cancer (*3*).

Glycans are synthesized in the endoplasmic reticulum and the Golgi apparatus by a complex interplay of glycosyltransferases—enzymes that add monosaccharides to a growing glycan chain—and various other enzymes and transporters involved in synthesis and delivery of donors and substrates needed for chemical reactions (*4*). However, our understanding of the mechanisms by which alternative glycosylation is achieved and regulated is very limited.

N-linked glycans attached to a protein can be released, and their abundance can be quantified using a range of analytical methods. These measurements represent a set of quantitative traits characterizing the glycome of the studied protein. As with any quantitative trait, the genetic determinants of the glycome can be studied by associating levels of glycans to polymorphic sequence variants measured in large cohorts of individuals [genome-wide association studies (GWAS)]. The first GWAS of the glycome identified associated variants in or near 7 glycosyltransferase genes and in 15 additional loci with no apparent role in protein glycosylation (*5*, *6*). The list was recently extended by six additional loci (*7*, *8*). However, there is still limited understanding of how these genes are functionally related or what their role is in the genetic regulation of IgG N-glycosylation in health and disease.

To address these questions, we performed the largest GWAS of the total IgG *N*-glycome to date, on 8090 samples from individuals of European ancestry, and more than doubled the number of associated loci. To prioritize genes with a plausible role in IgG glycosylation, we assessed whether variants were located in the coding regions of genes, showed pleiotropy with gene expression in biologically relevant cell types, and assessed enrichment in gene sets from different biological pathways. We explored how these genes are connected in a functional network and confirmed some of the network connections with in vivo functional follow-up. Last, we investigated pleiotropy with other complex traits and diseases by interrogating the overlap of glycosylation associations with susceptibility loci for other phenotypes. Where available, we also assessed whether IgG N-glycosylation, diseases, and gene expression are likely to be controlled by the same underlying causal variant using summary-level Mendelian randomization (SMR). This strategy not only resulted in the discovery of new candidate genes but also suggested how some of these genes could regulate glycosylation enzymes and how they could influence the aberrant glycosylation observed in diseases with an inflammatory signature.

## RESULTS

### Discovery and replication meta-analyses

We performed a discovery IgG N-glycosylation GWAS on four cohorts of European descent (*N* = 8090). Associations of 77 ultraperformance liquid chromatography (UPLC) IgG *N*-glycan traits with HapMap2 (release 22) imputed genetic data were studied. We assumed an additive linear model for each glycan trait, followed by a fixed-effect inverse-variance meta-analysis.

**Fig. 1:**
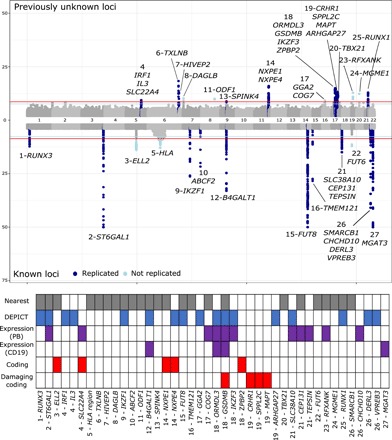
Gene prioritization in loci associated with IgG N-glycosylation. The Manhattan plot was created by taking the lowest *P* value at every genomic position from all 77 GWAS. For simplicity, the plot was trimmed at the equivalent of *P* = 10^−50^. The lowest observed *P* value in this analysis was 4.65 × 10^−276^ at *ST6GAL1*. Known loci, loci detected in previous IgG N-glycosylation GWAS; replicated, UPLC replication GWAS. Bottom: Summary of support for prioritization of every gene in the Manhattan plot. DEPICT, genes from enriched gene sets; expression, genes whose expression is pleiotropic with IgG N-glycosylation; CD19, B cells; PB, peripheral blood; coding, genes for which IgG N-glycosylation–associated SNP results in a changed amino acid sequence.

Overall, associations in 27 loci reached genome-wide significance [*P* ≤ 2.4 × 10^−9^; Bonferroni corrected for 21 independent glycan traits; (*6*)], and another 6 loci were suggestively significant (2.4 × 10^−8^ ≤ *P* < 2.4 × 10^−9^) (Table 1). Eight of the genome-wide significant loci confirmed previous findings from Lauc *et al.* (*6*), 5 confirmed those from Shen *et al.* (*7*) (one of which, *AZI1*, maps to the same locus as the suggestive association near *SLC38A10* from Lauc *et al.*), and 1 confirmed those from Wahl *et al.* (*8*) (Fig. 1 and table S1), while 14 loci were not previously associated with IgG glycosylation. Individual associations and Manhattan plots can be found in the online resource available at https://shiny.igmm.ed.ac.uk/igg_glycans_gwas/. For 19 of 27 significant loci (9 of 14 previously unassociated), the same single-nucleotide polymorphism (SNP)–glycan association was replicated in a meta-analysis of four independent European cohorts (*N* = 2388) with *P* ≤ 0.05/27 ≈ 1.9 × 10^−3^. None of the genes in novel loci have a known role in glycosylation. For all loci, the direction of effect estimates obtained for the UPLC data (effect either increasing or decreasing with the same allele) was the same in the discovery and the replication parts of the study (Table 1).

**Table 1:** Loci associated with IgG glycosylation. Each locus is represented by the SNP with the strongest association in the region. Locus, coded by “chromosome: locus start–locus end” (GRCh37); No. of SNPs, maximum number of SNPs independently contributing to trait variation; SNP, variant with the strongest association in the locus; position, position of the SNP on the NCBI (National Center for Biotechnology Information) build 37; EA, allele for which effect estimate is reported; OA, other allele; EAF, frequency of the effect allele; *R*^2^, sample size weighted average of imputation quality for the SNP with the strongest association in the locus; glycan, glycan associated with the reported SNP; No. of glycans, number of other glycans suggestively or significantly associated with variants in given locus; β, effect estimate for the SNP and glycan with the strongest association in the locus in the discovery; *P*, *P* value for the discovery effect estimate; β UPLC repl, effect estimate for the SNP and glycan with the strongest association in the locus in replication; *P* UPLC repl, *P* value for the effect estimate in replication. The loci replicated in UPLC replication at *P* ≤ 0.0019 are in bold. The loci from Lauc *et al.* (*6*), Shen *et al.* (*7*), and Wahl *et al.* (*8*) are in italics.

**Locus**	**Candidate** **genes**	**No. of** **SNPs**	**SNP**	**EA**	**OA**	**EAF**	***R*^2^**	**Glycan**	**No. of** **glycans**	**β**	***P***	**β UPLC** **repl**	***P* UPLC** **repl**
**Genome-wide significant**													
***1:25226001-25345011***	***RUNX3***	***1***	***rs10903118***	***T***	***C***	***0.484***	***0.886***	***IGP74***	***13***	***−0.121***	***5.14 × 10^−13^***	***−0.097***	***7.93 × 10^−4^***
***3:186712711-186738421***	***ST6GAL1***	***2***	***rs7621161***	***A***	***C***	***0.284***	***0.937***	***IGP29***	***27***	***−0.653***	***4.65 × 10^−276^***	***−0.682***	***1.55 × 10^−111^***
*5:95211647-95347786*	*ELL2*	*1*	*rs7700895*	*A*	*T*	*0.215*	*0.997*	*IGP35*	*4*	*0.155*	*1.20 × 10^−14^*	*0.097*	*4.61 × 10^−3^*
**5:131026218-131833599**	***IRF1-SLC22A4***	**1**	**rs11748193**	**A**	**T**	**0.399**	**0.987**	**IGP2**	**2**	**0.110**	**4.31 × 10^−10^**	**0.116**	**1.31 × 10^−4^**
***6:29299390-33883424***	*HLA* region	*2*	*rs3099844*	*A*	*C*	*0.136*	*0.995*	*IGP15*	*13*	*−0.227*	*1.12 × 10^−13^*	*−0.083*	*2.28 × 10^−1^*
**6:139617590-139636003**	***TXLNB***	**1**	**rs9385856**	**T**	**C**	**0.574**	**0.977**	**IGP70**	**24**	**0.151**	**5.05 × 10^−19^**	**0.111**	**1.54 × 10^−4^**
**6:143150223-143203591**	***HIVEP2***	**1**	**rs7758383**	**A**	**G**	**0.521**	**0.952**	**IGP13**	**3**	**0.125**	**9.61 × 10^−14^**	**0.225**	**1.12 × 10^−14^**
7:6520676-6537913	*DAGLB*	1	rs6964421	T	C	0.328	0.957	IGP14	7	0.117	5.31 × 10^−11^	0.000	9.95 × 10^−1^
***7:50325717-50361683***	***IKZF1***	***1***	***rs6421315***	***C***	***G***	***0.383***	***0.938***	***IGP62***	***37***	***0.192***	***4.70 × 10^−27^***	***0.133***	***8.97 × 10^−6^***
***7:150906453-150969535***	***ABCF2***	***1***	***rs7812088***	***A***	***G***	***0.119***	***0.981***	***IGP2***	***16***	***−0.250***	***2.06 × 10^−22^***	***−0.269***	***3.83 × 10^−9^***
8:103538266-103550211	*ODF1*	1	rs10096810	A	G	0.627	1.000	IGP77	4	−0.110	9.52 × 10^−11^	−0.074	1.37 × 10^−2^
***9:33041761-33186080***	***B4GALT1***	***3***	***rs10813951***	***A***	***G***	***0.732***	***0.967***	***IGP17***	***30***	***0.230***	***8.84 × 10^−34^***	***0.156***	***4.57 × 10^−6^***
**9:33205136-33375592**	***SPINK4***	**1**	**rs12341905**	**A**	**G**	**0.896**	**0.964**	**IGP53**	**4**	**0.168**	**1.46 × 10^−09^**	**0.199**	**4.99 × 10^−5^**
**11:114323627-114450529**	***NXPE1-NXPE1***	**1**	**rs481080**	**A**	**G**	**0.475**	**0.999**	**IGP29**	**3**	**−0.140**	**1.05 × 10^−16^**	**−0.099**	**5.28 × 10^−4^**
***14:65472891-66284991***	***FUT8***	***6***	***rs11847263***	***T***	***G***	***0.647***	***0.985***	***IGP42***	***16***	***−0.283***	***1.13 × 10^−58^***	***−0.252***	***5.93 × 10^−17^***
***14:105966019-106002352***	***TMEM121***	***1***	***rs4074453***	***T***	***C***	***0.758***	***0.996***	***IGP48***	***6***	***−0.218***	***3.82 × 10^−29^***	***−0.299***	***1.25 × 10^−13^***
16:23397113-23613191	*GGA2-COG7*	1	rs250555	T	C	0.860	0.961	IGP26	4	−0.155	6.76 × 10^−10^	−0.044	2.24 × 10^−1^
**17:37903731-38112190**	***ORMDL3-GSDMB-IKZF3-ZPBP2***	**1**	**rs7216389**	**T**	**C**	**0.487**	**0.999**	**IGP59**	**11**	**0.137**	**1.17 × 10^−15^**	**0.094**	**1.53 × 10^−3^**
**17:43463492-44896083**	***CRHR1-SPPL2C-MAPT-ARHGAP27***	**1**	**rs199456**	**T**	**C**	**0.196**	**0.984**	**IGP14**	**7**	**0.162**	**6.76 × 10^−14^**	**0.136**	**9.18 × 10^−4^**
**17:45518583-45874272**	***TBX21***	**1**	**rs11651000**	**A**	**G**	**0.150**	**0.981**	**IGP59**	**8**	**−0.165**	**2.66 × 10^−12^**	**−0.144**	**4.05 × 10^−4^**
***17:79165171-79257880***	***SLC38A10-CEP131-TEPSIN***	***1***	***rs2725391***	***T***	***C***	***0.460***	***0.974***	***IGP24***	***12***	***0.133***	***9.91 × 10^−16^***	***0.177***	***1.15 × 10^−9^***
*19:5822316-5845974*	*FUT6*	*1*	*rs874232*	*T*	*C*	*0.565*	*0.971*	*IGP12*	*5*	*0.119*	*7.85 × 10^−13^*	*0.070*	*2.08 × 10^−2^*
19:19260586-19296217	*RFXANK*	1	rs7257072	T	C	0.515	0.997	IGP9	12	−0.122	1.59 × 10^−13^	−0.068	1.97 × 10^−2^
20:17818141-17833534	*MGME1*	1	rs2745851	A	G	0.376	0.982	IGP38	10	−0.125	4.61 × 10^−13^	−0.094	8.82 × 10^−3^
**21:36546756-36665202**	***RUNX1***	**1**	**rs7281587**	**A**	**G**	**0.247**	**0.998**	**IGP45**	**19**	**−0.144**	**1.13 × 10^−13^**	**−0.171**	**7.51 × 10^−7^**
***22:24093789-24182500***	***SMARCB1-DELR3-CHCHD10-VPREB3***	***1***	***rs17630758***	***A***	***G***	***0.152***	***0.991***	***IGP66***	***25***	***−0.308***	***1.20 × 10^−41^***	***−0.386***	***4.94 × 10^−23^***
***22:39774448-39860868***	***MGAT3***	***1***	***rs5750830***	***A***	***C***	***0.740***	***0.965***	***IGP40***	***28***	***−0.344***	***7.74 × 10^−69^***	***−0.363***	***5.26 × 10^−29^***
**Suggestive**													
1:246854862-246963137	LINC01341	1	rs3795464	A	G	0.383	0.984	IGP73	2	−0.099	1.01 × 10^−08^		
2:100636757-100805273	AC092667.1	1	rs2309748	A	T	0.647	0.993	IGP34	2	−0.105	3.38 × 10^−09^		
17:16820099-16875636	TBC1D27-TNFRSF13B	1	rs4561508	T	C	0.105	0.975	IGP9	2	0.163	5.70 × 10^−09^		
17:56398006-56410041	MIR142	1	rs2526378	A	G	0.549	0.935	IGP31	1	−0.100	4.44 × 10^−09^		
19:1614910-1658699	TCF3	1	rs4807942	T	C	0.878	0.661	IGP68	1	0.154	1.54 × 10^−08^		
20:52170177-52212273	ZNF217	1	rs1555926	T	C	0.789	0.992	IGP11	3	−0.118	6.96 × 10^−09^		

To further validate our findings, we analyzed all significantly associated SNPs in a cohort where glycans were measured with liquid chromatography coupled to electrospray mass spectrometry (LCMS; *N* = 1842). From the 27 genome-wide significant SNPs, 17 were associated with at least one of the LCMS-measured IgG glycopeptide levels at Bonferroni-corrected *P* ≤ 3.7 × 10^−5^. Of these 17, 14 loci were also replicated in the UPLC replication study (table S2).

We performed an approximate conditional and joint analysis using GCTA-COJO (genome-wide complex trait conditional and joint analysis) software (*9*) to investigate secondary associations in the 27 loci. We found evidence of multiple SNPs independently contributing to glycan level variation for four loci. Three of these loci span glycosyltransferase genes, coding for enzymes directly involved in biosynthesis of glycans (*4*) (table S3). The greatest number of independently associated SNPs (six) was observed for the fucosyltransferase locus, *FUT8*, followed by three SNPs in the galactosyltransferase locus, *B4GALT1*, and two SNPs in the sialyltransferase gene, *ST6GAL1*. Two SNPs in the fourth locus, spanning the human leukocyte antigen (HLA) region on chromosome 6, are likely to be due to the complexity of this region, where multiple antigen-presenting genes are in close proximity and in high linkage disequilibrium (LD).

Using only the marginal effects of top SNPs, we were able to explain up to 20% of the genetic variance (for a single glycan trait). Uncovering additional variants in the conditional analysis resulted in a maximum of 22% variance explained. These maxima were reached for IGP29, the percentage of monosialylation of fucosylated digalactosylated structures without bisecting *N*-acetylglucosamine, GlcNAc (table S3).

### Prioritizing genes associated with IgG N-glycosylation

We applied the following strategy to prioritize the most likely functional genes. The Data-driven Expression Prioritized Integration for Complex Traits (DEPICT) framework (*10*) was used to perform a gene set and tissue/cell type enrichment analysis and to provide additional evidence for gene prioritization. Next, for all candidate genes in every locus, we explored evidence for genes containing SNPs affecting amino acid sequence using the Variant Effect Predictor (VEP) (*11*) and genes whose expression is likely to be regulated by the same underlying variant as N-glycosylation of IgG. For the latter, we applied SMR with heterogeneity in dependent instruments (HEIDI) (*12*) on expression quantitative trait loci (eQTLs) from five different immune cells (neutrophils, macrophages, B cells, and two types of T cells) from the CEDAR dataset [*N* = 350; Momozawa *et al.* (*13*)] and peripheral blood from Westra *et al.* (*14*) (*N* = 5311). This test assesses whether the coassociation of two traits to the same region may be due to pleiotropic action of the same variants to both traits or due to the variant being in LD with two independent causal variants, each exhibiting independent effects on each trait. More specifically, a pleiotropic scenario would assume that the same unobserved variant is responsible for the association with both gene expression and glycan levels, while an LD scenario would assume that coassociation is due to two or more underlying variants that are in LD and independently contribute to either gene expression or glycan levels.

The DEPICT gene prioritization tool provided evidence of prioritization for 18 genes in 14 loci at false discovery rate (FDR) <0.05 and another 3 genes at FDR <0.2 (Supplementary Note, Appendix Table 9). In four genes, associated variants were predicted to result in a potentially deleterious amino acid change, and in five genes, probably benign amino acid changes. SMR/HEIDI indicated that IgG N-glycosylation–associated variants in 9 loci had pleiotropic effects on expression of 13 genes in different tissues, including B cells (4 genes in 3 loci), the cell lineage responsible for IgG synthesis. For 11 loci, we did not find any additional evidence to prioritize target genes and report the gene closest to the strongest association in the region (Fig. 1 and the Supplementary Note; https://shiny.igmm.ed.ac.uk/igg_glycans_gwas/).

To obtain insights into the biological pathways that these genes are involved in, we performed FUMA’s GENE2FUNC (*15*) for Gene Ontology (GO) and DEPICT for gene set enrichment analyses. In total, our prioritized genes were significantly enriched in 75 different GO gene sets. These gene sets reflect three higher-level biological processes: glycosylation (23 gene sets), immune system processes (22 gene sets), and transcription (7 gene sets) (table S4). DEPICT tests for enrichment in preconstructed gene sets that are based on information from protein-protein interactions (PPIs), molecular and biochemical pathways, gene coexpression, and gene sets based on mouse gene knockout studies. No gene sets were enriched at FDR <0.05, but 397 gene sets were enriched at FDR <0.2. These gene sets are connected to various processes involved in immunity, B cell life cycle, and antibody production and quantity (table S5).

### Functional network of loci associated with IgG N-glycosylation

Next, we investigated how genes in associated loci are functionally connected with each other. We assume that two loci with a similar glycome-wide effect (effect of the top SNP in the locus on all glycan traits) are likely to have a similar role in the regulation of glycosylation and may be a part of the same biological pathway. Our assumption is based on the following. We analyze the glycosylation of a single protein (IgG) and determine 77 different glycan traits. These traits arise as a result of the activity of different enzymes in the glycosylation biosynthesis pathway. It can be expected that changing the rate of a specific reaction would lead to specific changes of glycan profile, regardless of the specific mechanism (e.g., alteration in substrate availability, lower activity of enzyme as a result of a mutation changing its structure, a regulatory variation changing the level of expression of a gene encoding relevant enzyme), leading to the rate change. For example, it can be expected that a regulatory variant that is associated with expression levels of glycosyltransferase gene *MGAT3* [mannosyl (β-1,4-)-glycoprotein β-1,4-*N*-acetylglucosaminyltransferase] will have an influence on all glycan traits that contain bisecting GlcNAc. This could be a variant changing the structure of this acetylglucosaminyltransferase, a regulatory variant in the promoter of the *MGAT3* gene, a variant disrupting an enhancer of *MGAT3*, or it could be a regulatory variant that changes expression of a transcription factor (TF) binding to the enhancer of *MGAT3*. Either way, the rate of reaction controlled by the product of *MGAT3* would change, leading to a similar effect on all glycan traits that contain bisecting GlcNAc.

To create specific hypotheses regarding the regulatory functions of our loci, we defined the glycome-wide effect of an SNP as a vector of effects [in the form of *z* scores, where *z* score = β/*se*(β)] of the given SNP on each of the glycans. As can be seen in fig. S1, some loci have notably similar effects on all glycan traits. We therefore constructed a functional network by computing Spearman pairwise correlations of glycome-wide effects of all 27 SNPs that tag each locus. Given that the direction of the GWAS estimates depends on the allele used as a reference, the sign of correlation is not informative in this analysis. Therefore, we report absolute values of correlation coefficients. Although nodes in this network represent effects of top SNPs, we marked nodes by using the names of candidate genes in each locus, and we used these names to denote loci throughout the text. Below, we focus our discussion only on clusters that contain glycosyltransferases (*MGAT3*, *FUT8*, *B4GALT1*, and *ST6GAL1*), because these enzymes catalyze the transfer of monosaccharides to a growing glycan chain and therefore have a known and well-established role in the biosynthesis of glycans. As outlined above, we hypothesize that any locus with a similar glycome-wide effect to a glycosyltransferase locus is likely to either regulate the expression levels of the enzyme or modulate its activity through other indirect effects.

To validate the functional network, we applied both computational and experimental approaches. We first pruned the correlation network for significant correlations [*P* ≤ 0.05/(27 × 26)/2 ≈ 1.4 × 10^−4^] and performed permutation analysis with 100,000 random SNPs. We also compared our network with the STRING human PPI database (*16*).

**Fig. 2:**
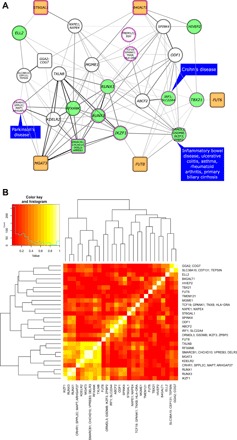
Functional network of loci associated with IgG N-glycosylation. Correlation estimates are computed on the basis of squared pairwise Spearman’s correlation of SNP effects. The loci are denoted with names of genes that were prioritized in regions tagged by the given lead SNP. (**A**) Functional network of loci associated with IgG N-glycosylation. In this network, each node represents a lead SNP in the locus, and each edge represents the squared correlation of glycome-wide effects of the two nodes. Only significant correlations after multiple testing correction (*P* ≤ 1.4 × 10^−4^) are shown. The thickness and intensity of edges depend on variation in one locus explained by the effect estimates in the second locus. Round-edged rectangular nodes denote genes that are, according to GO, involved in glycosylation; purple-edged nodes denote genes involved in immune system processes; green nodes denote loci containing genes involved in transcription regulation; orange nodes denote glycosyltransferases; and blue rectangles indicate diseases pleiotropic with IgG glycans in the given locus (Table 3). (**B**) Hierarchical clustering of pairwise Spearman’s locus-effect correlations.

The strongest overall correlation, a Spearman’s ρ of 0.97, was observed between IgG–*N*-glycome–wide effects of the top SNP in the *MGAT3* locus and the top SNP in the *SMARCB1-DELR3-CHCHD10-VPREB3* locus (Fig. 2A). None of the randomly selected SNPs exhibited such a strong correlation with these two loci in the permutation analysis (Table 2 and table S6). Recent work by Sharapov *et al.* (*17*) on genetics of the total plasma proteome N-glycosylation, of which IgG is a major part, suggested that the likely candidate gene is *DERL3*. *DERL3* encodes a protein involved in endoplasmic reticulum–associated degradation for misfolded luminal glycoproteins. Data provided in this study shift the balance of evidence toward the *SMARCB1*, as it is unlikely that modulation of glycoprotein degradation would result in effects similar to those observed when perturbing MGAT3.

**Table 2:** In silico evidence for edges from functional network of IgG N-glycosylation loci. Permutation analysis was used to assess the distribution of glycome-wide SNP effect pairwise correlations and the STRING PPI database (*16*) to search for biological validation of observed links. All reported correlations are statistically significant after Bonferroni correction. Corr, Spearman’s correlation coefficient of *z* scores of SNP1 and *z* scores of SNP2; quant, quantile of distribution at which the given ρ2 was observed in permutation analysis; STRING score, probability that the link exists obtained by combining evidence from different sources. SNPs that were replicated in UPLC replication are in bold.

**SNP1**	**SNP2**	**Locus 1**	**Locus 2**	**Corr**	**SNP 1 quant**	**SNP 2 quant**	**STRING** **gene 1**	**STRING** **gene 2**	**STRING** **score**
**Glycosyltransferase loci**									
**rs5750830**	**rs17630758**	*MGAT3*	*SMARCB1-DERL3-CHCHD10-VPREB3*	0.967	0	0			
**rs10813951**	**rs12341905**	*B4GALT1*	*SPINK4*	0.805	3.54 × 10^−3^	7.72 × 10^−3^			
**rs10813951**	**rs7758383**	*B4GALT1*	*HIVEP2*	0.711	1.87 × 10^−2^	3.83 × 10^−3^			
**rs7621161**	rs481080	*ST6GAL1*	*NXPE1; NXPE4*	0.773	6.00 × 10^−5^	3.01 × 10^−3^			
**rs7621161**	rs7700895	*ST6GAL1*	*ELL2*	0.581	8.32 × 10^−3^	2.45 × 10^−2^			
**rs11847263**	**rs7216389**	*FUT8*	*ORMDL3-GSDMB-IKZF3-ZPBP2*	0.709	8.42 × 10^−3^	2.59 × 10^−2^			
**rs11847263**	**rs6421315**	*FUT8*	*IKZF1*	0.624	2.62 × 10^−2^	9.69 × 10^−2^			
**Other loci**									
**rs10903118**	**rs7281587**	*RUNX3*	*RUNX1*	0.891	2.00 × 10^−5^	3.00 × 10^−5^	*RUNX3*	*RUNX1*	0.9
**rs7281587**	**rs17630758**	*RUNX1*	*SMARCB1-DERL3-CHCHD10-VPREB3*	0.597	3.25 × 10^−2^	1.59 × 10^−2^	*RUNX1*	*SMARCB1*	0.4
**rs10903118**	**rs17630758**	*RUNX3*	*SMARCB1-DERL3-CHCHD10-VPREB3*	0.694	1.15 × 10^−2^	3.33 × 10^−3^			
**rs7216389**	**rs6421315**	*ORMDL3-GSDMB-IKZF3-ZPBP2*	*IKZF1*	0.609	9.57 × 10^−3^	2.49 × 10^−2^	*IKZF3*	*IKZF1*	0.42
**rs7216389**	**rs6421315**	*ORMDL3-GSDMB-IKZF3-ZPBP2*	*IKZF1*	0.609	9.57 × 10^−3^	2.49 × 10^−2^	*ZPBP2*	*IKZF1*	0.48
**rs7281587**	**rs6421315**	*RUNX1*	*IKZF1*	0.626	2.56 × 10^−2^	2.15 × 10^−2^	*IKZF1*	*RUNX1*	0.6
**rs11748193**	rs3099844	*IRF1-SLC22A4*	*TCF19-GPANK1-TNXB-HLA-DRA*	0.231	8.82 × 10^−2^	9.86 × 10^−2^	*HLA-DRA*	*IRF1*	0.93
**rs7281587**	**rs11748193**	*RUNX1*	*IRF1-SLC22A4*	0.283	1.63 × 10^−1^	6.02 × 10^−2^	*RUNX1*	*SLC22A4*	0.7
rs7257072	rs3099844	*RFXANK*	*TCF19-GPANK1-TNXB-HLA-DRA*	0.23	1.77 × 10^−1^	9.85 × 10^−2^	*HLA-DRA*	*RFXANK*	0.62

The second strongest association, a Spearman’s ρ of 0.94, was between lead SNPs in TFs *RUNX3* and *RUNX1* loci. This link was validated in the permutation analysis and observed in the STRING PPI network. *RUNX3* and *RUNX1* loci were also strongly correlated with both the *MGAT3* and *SMARCB1-DELR3-CHCHD10-VPREB3* loci (Table 2). A proxy for the top SNP in the *MGAT3* locus, SNP rs8137426 (LD *R*^2^ = 1 with rs5750830), is located in a region bound by TF RUNX3, although not directly within its binding motif (fig. S2A). In our SMR/HEIDI analysis, we found evidence of the same association having a pleiotropic effect on both *MGAT3* expression in CD19^+^ B cells and IGP40, a glycan trait related with bisecting GlcNAc (https://shiny.igmm.ed.ac.uk/igg_glycans_gwas/). This suggests that the associated SNP could influence binding of TFs (RUNX1 or RUNX3), which, together with the chromatin remodeling protein SMARCB1, regulate expression of *MGAT3*, resulting in an increased incidence of bisecting GlcNAc in all fucosylated disialylated structures of IgG (IGP40; Table 1).

The *FUT8* locus had the strongest glycome-wide association with the *ORMDL3-GSDMB-IKZF3-ZPBP2* and *IKZF1* loci (ρ = 0.71 and 0.62). The latter two loci also show highly similar glycome-wide effects with each other (ρ = 0.78) and were observed as interacting in the STRING PPI network (Table 2 and table S7).

Two remaining galactosyltransferases had the strongest correlations with loci where the underlying hypothesis of regulation is less straightforward. The most strongly correlated loci with the galactosyltransferase *B4GALT1* locus were *SPINK4* and *HIVEP2* (ρ = 0.8 and 0.71). Top SNPs from the *NXPE1-NXPE4* and *ELL2* loci had the most similar glycome-wide effects to the top SNP from the sialyltransferase *ST6GAL1* locus (ρ = 0.77 and 0.58). Full results of the permutation analysis and STRING PPI are available at the online resource (https://shiny.igmm.ed.ac.uk/igg_glycans_gwas/).

Last, we assessed whether there is evidence of a subnetwork of genes regulating each specific glycosyltransferase by performing hierarchical clustering on the glycome-wide SNP effect correlation matrix. We observed three clusters: the cluster with *MGAT3*, the cluster with *FUT8*, and the cluster with *B4GALT1* and *ST6GAL1.* Several loci containing genes coding for TFs cluster together with the *MGAT3* locus, suggesting a complex regulatory network for this glycosyltransferase gene (and/or for the factors regulating this gene, i.e., RUNX1 is repressed by RUNX3) (Fig. 2B).

To further explore this possibility, we analyzed whether IgG glycosylation–associated SNPs were predicted to disrupt binding sites of TFs encoded by genes from this network more often than by chance. To assess the influence of glycosylation SNPs on binding of TFs from our network (IKZF1, IKZF3, RUNX1, RUNX3, IRF1, SMARCB1, and TBX21), we used Regulatory Sequence Analysis Tools (*18*). Briefly, if an associated SNP maps to the conserved position in the TF-binding motif and its effect allele is different than the conserved allele at that position, the SNP will be predicted to strongly affect binding of the TF. Alternatively, the allele can also completely disrupt or create the binding motif, resulting in a loss of a known or introduction of a new TF-binding site. In four loci spanning the glycosyltransferase genes, the associated SNPs resulted in either introduction or disruption of binding sites for TFs RUNX1, RUNX3, IKZF1, SMARCB1, TBX21, and IRF1. We observed the same disruption/introduction of TF-binding sites for all these TFs too and therefore confirmed some well-known regulation feedback loops between TFs RUNX1 and RUNX3 and IKZF1 and IKZF3 (table S8) (*19*, *20*). To assess whether these TF-binding site alterations are specific for associated SNPs, we compared the frequency of TF-binding alterations of associated and nonassociated SNPs. In seven glycosylation loci, the associated SNPs were at least two times more likely to affect binding of TF than nonassociated SNPs from the same region. The highest difference in the number of SNPs affecting the TF-binding site was observed for *FUT8*, *MGAT3*, and loci coding for TFs that are members of their cluster. Associated SNPs from the *FUT8* and *MGAT3* loci have a significant effect on binding of TF from our network, while nonassociated SNPs from their proximity have no effect on binding (table S9). In summary, we not only found TFs associated with IgG glycosylation but also showed that glycosylation-associated SNPs can potentially alter their binding in other glycosylation-associated loci. These results not only reinforce some of the links suggested in our network but also confirm some known feedback loops between different TFs involved in B cell differentiation and biology (e.g., interactions of IKZF1 and IKZF3 and RUNX1 and RUNX3) (*19*, *20*).

### Experimental validation of the functional links between *IKZF1* and *FUT8*

**Fig. 3:**
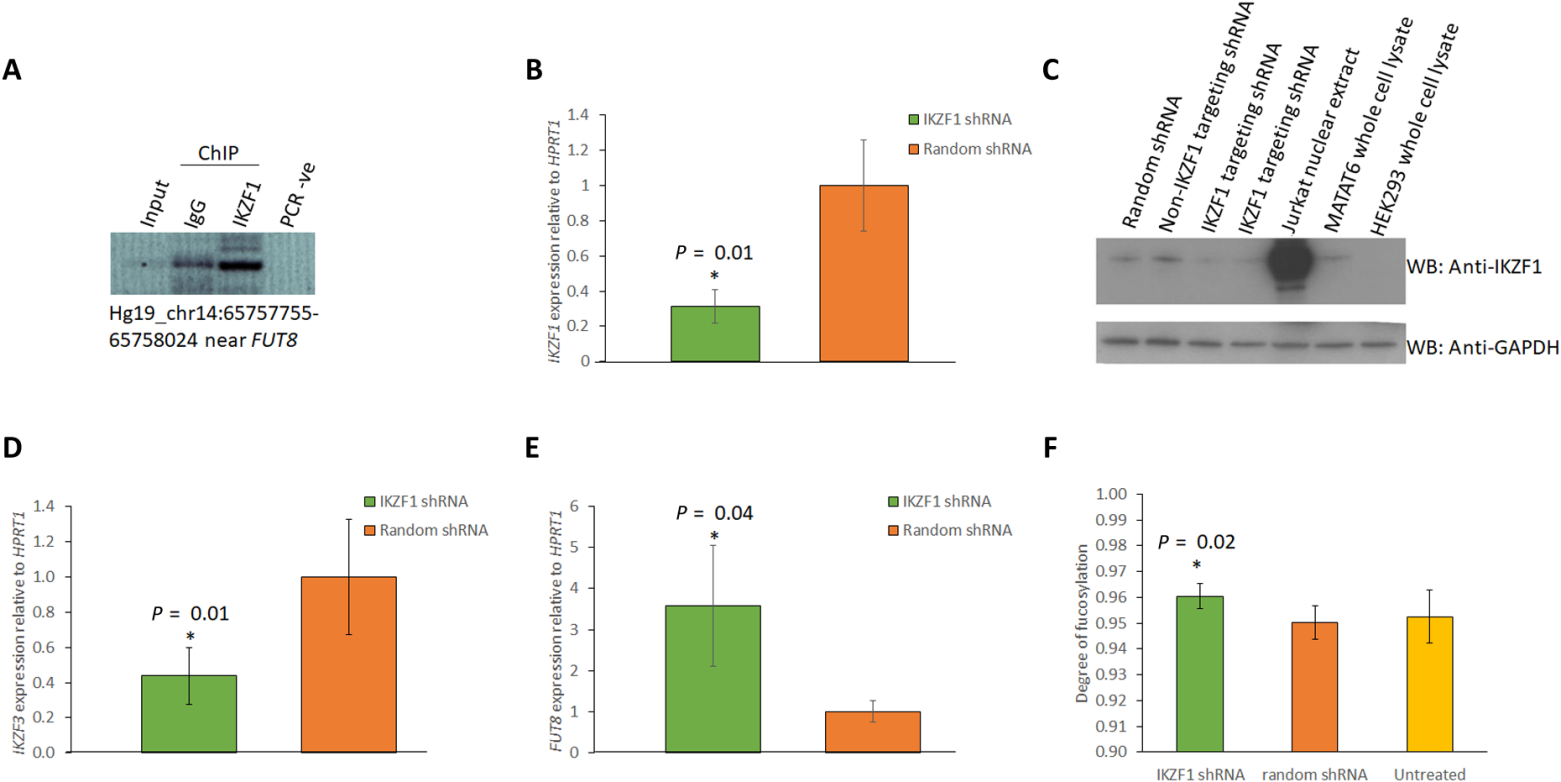
Results of in vivo validation of the functional links between *IKZF1* and *FUT8.* (**A**) IKZF1 binds to a regulatory region upstream of *FUT8*. (**B**) Knockdown of *IKZF1* leads to decreased expression of *IKZF1* (*n* = 3). (**C**) Representative Western blot showing that *IKZF1* is depleted at the protein level (protein reduced by around 50% compared with a random shRNA line or a non-IKZF1 targeting shRNA line). WB, Western blot. (**D**) Knockdown of *IKZF1* leads to decreased expression of *IKZF3* (*n* = 3). (**E**) Knockdown of *IKZF1* leads to increased expression of *FUT8* in the LCL (*n* = 3). (**F**) There is a small but significant increase in fucosylation of IgG secreted by the LCLs in which *IKZF1* is knocked down (*n* = 3 lines, measured at two time points). All *P* values are *t* tests, and error bars show SD from the mean. Asterisk indicates *P* value <0.05.

Our network-based approach allowed us to show that variants in or near *IKZF1* and *IKZF3* share glycome-wide similarities with the top SNP in the *FUT8* locus. We hypothesized that the TFs IKZF1 and IKZF3 may regulate the expression of *FUT8*. We further observed notable pleiotropy between variants in the *IKZF3-ORMDL3-GSDMB-ZPBP2* locus with inflammatory conditions including inflammatory bowel disease (IBD), ulcerative colitis (UC), and rheumatoid arthritis (RA) (Fig. 2). We therefore aimed to disrupt this node of our network in our cellular model. Microarray and RNA sequencing data from the lymphoblastoid cell line (LCL) used in this study showed that *IKZF1* was more highly expressed than *IKZF3*. Because these genes are known to regulate one another, we decided to target *IKZF1*. To investigate the potential link between *IKZF1* and *FUT8*, we identified IKZF1-binding sites on chromosome 14 near *FUT8* in the LCL GM12878 Encode chromatin immunoprecipitation sequencing (ChIP-seq) data. We performed ChIP followed by polymerase chain reaction (PCR) to confirm IKZF1 binding at one of these sites in an IgG-secreting human B cell–derived LCL, MATAT6 (Fig. 3A). We then investigated the role of IKZF1 in the regulation of *FUT8* by conducting stable short hairpin RNA (shRNA)–mediated knockdown of *IKZF1* using shRNA in MATAT6 cells. This resulted in depletion of the *IKZF1* transcript and protein (Fig. 3, B and C). Knockdown of *IKZF1* was accompanied by a significant down-regulation of *IKZF3* (Fig. 3D) and a 3.5-fold up-regulation in the expression of *FUT8* (*t* test, *P* = 0.04; Fig. 3E), corroborating a link between *IKZF1* and *FUT8* and highlighting the dynamic relationships between TFs in B cells. Next, we looked at changes in the glycosylation of secreted IgG in cells with depleted IKZF1 at two different time points and observed a small but significant (*P* = 0.02; table S10) increase in fucosylation (Fig. 3F) resulting from *IKZF1* knockdown. While this change is small, the low SE of triplicate measurements and independent replication of the experiment (second time point) demonstrate its robustness (table S10). In addition, one must consider that most of the glycans are fucosylated and that further fucosylation might be limited by specificities of glycosyltransferases involved in the process—the addition of bisecting GlcNAc to a growing glycan chain by MGAT3 inhibits fucosylation of these glycans (*21*). This change also results in a 20% decrease (from 5 to 4%) in afucosylated glycans [glycoforms activating antibody-dependent cellular cytotoxicity (ADCC)]. These data are consistent with decreased expression of *IKZF1* resulting in increased *FUT8* expression and, thus, increased IgG fucosylation. Hence, although there is no in silico evidence that the lead SNP in the *FUT8* locus overlaps with a predicted IKZF1-binding motif, and there is no evidence for the associated SNP being a *FUT8* eQTL, we show that SNPs that are in LD with the lead *FUT8* SNP can alter binding of *IKZF1* (table S8). Furthermore, this TF can bind to regulatory regions of *FUT8* and may act cooperatively with additional factors to modify its expression. To explore this further, we used publicly available Hi-C profiling performed at 1-kb resolution in the LCL GM12878. These data indicate that SNPs in the *FUT8* locus lie in the same chromosomal topologically associating domain as the transcription start site of *FUT8* (fig. S2B). CCCTC-binding factor (CTCF) plays a crucial role in maintaining the three-dimensional structure of the genome, and disruption of CTCF binding by SNPs in LD with rs4400971 may modify the interactions formed between enhancer and promoter regions of *FUT8* to affect expression of the glycosyltransferase enzyme (for details, see Supplementary Note).

### Pleiotropy with complex traits and diseases

Because aberrant glycosylation patterns have been observed in various diseases (*22*–*24*), we explored pleiotropy between the glycosylation loci and other traits. We first searched for whether suggestive or significant glycosylation-associated SNPs and their proxies (*P*_GWASglyco_ ≤ 2.4 × 10^−8^; LD *R*^2^ ≥ 0.8) were previously reported as associated (*P*_GWAStraits_ ≤ 5 × 10^−8^) with other traits using Ensembl’s BioMart tool (*25*). Overall, 219 SNPs from 16 glycosylation loci were reported to be associated with 83 other traits in various databases, 47 of which map outside the HLA region (chr6:29570005-33377657, GRCh37) (https://shiny.igmm.ed.ac.uk/igg_glycans_gwas/).

To expand on the findings from Lauc *et al.* (*6*), where they report that some variants in LD with glycosylation variants are pleiotropic with diseases, we tested whether these coassociations may be due to the same underlying causal variant (pleiotropic) or are suggestive of LD between distinct causal variants each controlling different traits. We performed the SMR/HEIDI test on traits for which the full summary-level GWAS data were available. We excluded the HLA region from our analysis because of its complex LD structure, which is incompatible with this method’s sensitivity to LD. In total, we analyzed 10 traits (see details in Supplementary Note) and 55 glycan-trait combinations.

In three glycosylation loci, we found evidence for pleiotropy (*P*_SMR_ ≤ 9.1 × 10^−4^, *P*_HEIDI_ ≥ 0.05) between at least one IgG glycan and eight other diseases or traits [Crohn’s disease (CD), IBD, UC, RA, primary biliary cirrhosis (PBC), asthma, high-density lipoprotein (HDL) cholesterol, and Parkinson’s disease (PD)] and expression of *GSDMB* and *ORMDL3* in B and T cells and *IKZF3* and *SLC22A4* in peripheral blood. In two glycosylation loci, SMR/HEIDI analysis was more suggestive of LD rather than a pleiotropy scenario (*P*_HEIDI_ ≤ 0.05), where different variants in the same locus are suggested to be causal for glycan levels and height and ankylosing spondylitis (Table 3). Full results of the SMR/HEIDI test for complex traits can be found in the online resource https://shiny.igmm.ed.ac.uk/igg_glycans_gwas/.

**Table 3:** Summary of the HEIDI testing for pleiotropy versus LD between variants coassociated with IgG glycan levels, complex traits, and gene expression. All traits have significant (*P*_SMR_ ≤ 9.1 × 10^−4^) coassociation. In these loci where the associated variant was the same in IgG glycosylation, complex traits, and gene expression, we report only glycans pleiotropic with both expression and complex traits. The HEIDI test distinguishes between pleiotropy (shared causal variant, HEIDI *P* ≥ 0.05) or causal variants in LD (HEIDI *P* < 0.05). Glycan trait descriptions: G, galactose; F, fucose; N, bisecting GlcNAc; S, sialic acid; gene expression: CD4 and CD8, helper and cytotoxic T cells; CD19, B cells; PB, peripheral blood.

**Locus**	**IGP**	**Glycan description**	**Complex traits**	**SMR direction**	**Gene** **expression**	**SMR direction**
**Shared causal variant (pleiotropy)**						
IRF1-SLC22A4	IGP2 and IGP42	G0	CD	+	*SLC22A4* (PB)	−
ORMDL3- GSDMB- IKZF3-ZPBP2	IGP2, IGP6, IGP42, and IGP46	G0 and G1	Asthma, HDL, PBC, IBD, RA, and UC	+	*ORMDL3* (CD4, CD8, CD19, and PB)	−
					*GSDMB* (CD19 and PB)	−
					*IKZF3* (PB)	+
	IGP58, IGP59, IGP60, and IGP61	% Fucosylation in total and in agalactosylated/ monogalactosylated/ digalactosylated glycans	Asthma, PBC, HDL, RA, and UC	−	*ORMDL3* (CD4, CD8, CD19, and PB)	+
					*GSDMB* (CD19 and PB)	+
					*IKZF3* (PB)	−
CRHR1-SPPL2C- MAPT- ARHGAP27	IG14, IGP49, and IGP54	G1FN, G2FN, % fucosylation of G1 glycans	PD	−		
**Distinct causal variants in LD**						
RUNX3	IGP65, IGP69, IGP74, IGP75, and IGP76	Ratio of fucosylated digalactosylated structures	Ankylosing spondylitis			
IRF1-SLC22A4	IGP2 and IGP42	G0	Height and IBD			

## DISCUSSION

A recent study of the IgG glycome in 95 mouse strains from the collaborative cross cohort revealed that differences in glycosylation make IgG structure quite diverse between different strains (*26*). Despite the absence of a direct genetic template for individual glycans, these differences are heritable, indicating that glycoprotein structure is being inherited as a complex trait. The main enzymes involved in protein N-glycosylation are well characterized (*4*), but the genetic regulation of the process of glycosylation is poorly understood. To address this, we performed the largest GWAS of the IgG glycome to date, identifying 27 loci and therefore doubling the number of previously associated loci. While 10 of these loci contain known glycosylation enzymes and other previously associated genes, 14 novel loci contain genes with no known role in glycosylation. We were able to explain as much as 22% of variance in glycan levels (for the degree of monosialylation of fucosylated digalactosylated structures without bisecting GlcNAc, IGP29). Compared to previous studies, we do not just report the gene closest to the strongest association signal but have performed detailed analyses to seek the most biologically relevant annotation of our associated loci. Our candidate genes either have associated variants located in the coding regions of genes, show pleiotropy with gene expression in biologically relevant cell types, or are enriched in gene sets from appropriate biological pathways. We also explored how these genes are connected in a functional network, proposing mechanisms of the regulation of known glycosylation enzymes, and confirmed some of the network connections with in vitro functional follow-up.

As previously observed for GWAS of other complex traits, SNPs associated with IgG glycosylation predominantly map to noncoding regions of the genome, suggesting that regulatory variation at distal enhancers influences interindividual differences in glycan profiles. Several lines of evidence support this: the association of variants in or near genes coding for TFs, a high incidence of associated variants that are predicted to affect binding of those TFs, and pleiotropy between variants regulating the expression of the glycosyltransferases in B cells and IgG N-glycosylation. In addition, missense variants were observed only in genes without a previously known role in glycosylation and not in any glycosyltransferase genes where damaging mutations are known to result in severe disease (*27*). IgG is synthesized and secreted by terminally differentiated B cells called plasma cells. Gene set enrichment analysis of our GWAS loci identified enrichment not only for GO terms associated with glycosylation and processes involved in the differentiation and maturation of B cells but also for GO terms associated with TF activity. This suggests that IgG N-glycosylation is more likely to be regulated at the level of expression of the main enzymes, their intracellular localization, or substrate availability than by impaired protein function.

We also capitalized on the omics setting of this study to create a data-driven network of the associated loci, suggesting mechanistic hypotheses on how new loci may influence known glycosyltransferases. We then used in vitro studies to validate selected findings. Within the network, most of the nodes clustered around a group of TFs known to have central roles in B cell maturation and differentiation: RUNX1 (*28*), RUNX3, IKZF1 (*29*), and IKZF3 (*30*). This clustering supports a TF-driven regulatory network acting in B lymphocytes to affect the expression of key glycosyltransferase enzymes. We hypothesized that associated SNPs would alter TF binding. In silico assessment in LCLs showed that glycosylation-associated SNPs alter binding of glycosylation-associated TFs in associated glycosyltransferase loci, as well as being involved in the regulation of TFs themselves. We identified seven loci as containing associated SNPs for which one allele disrupts a binding motif for at least one TF in our network, two or more times more frequently than nonassociated SNPs from the same region. This is consistent with the identification of functionally important variants (or in LD with) in cis-regulatory elements acting to alter gene expression. For example, in our network, we suggest that a number of TFs and chromatin remodeling proteins regulate expression of *MGAT3*, the enzyme that transfers a GlcNAc to the mannose core of N-linked oligosaccharides to produce bisecting GlcNAc glycan structures. We have provided in silico evidence that associated SNPs from the *MGAT3* region disrupt binding sites for these TFs and are pleiotropic with *MGAT3* expression in CD19^+^ B cells. In our network, we also observed a link between *IKZF1* and *FUT8.* We provide direct evidence that IKZF1 transcriptionally regulates *FUT8*, showing, first, that IKZF1 binds to regulatory regions of *FUT8* and, second, that *IKZF1* knockdown results in increased *FUT8* expression and increased IgG fucosylation. We also observed a significant reduction in the expression of *IKZF3* when IKZF1 was depleted, consistent with these TFs regulating one another (*20*) and explaining the similarity of their glycome-wide effects. The mechanism underlying the strong correlation between the glycome-wide effects of variants at other loci is less clear, highlighting the challenges in interpreting the effects of noncoding variants. Further work is required to determine exact mechanisms, although disruption of structural interactions or context-specific effects to stimuli is likely to be important.

Using glycosyltransferases as a positive control for gene prioritization, it is apparent that none of the methods used are sufficient in isolation, highlighting the complementarity and limitations of different approaches. In current databases and pathways, there is no information on the regulation of glycosyltransferases, so complementing GWAS with data-driven network analysis, as performed here, can provide useful insights into potential mechanisms regulating genes of interest.

Aberrant glycosylation profiles were in the past observed in many diseases. An increase in agalactosylated structures is related to the proinflammatory effects of IgG (*31*). The absence of the core fucose is associated with increased ADCC (*2*, *32*). Previous studies reported that some IgG glycosylation–related SNPs were also associated with a range of autoimmune diseases and hematological cancers (*6*, *7*), but the mechanisms are still mostly unknown. We explored pleiotropy with complex traits and diseases and showed that diseases having the same associated SNPs are enriched in immune system disorders. Some of these diseases are also likely to have the same underlying causal variants as IgG N-glycosylation. In the *ORMDL3-GSDMB-IKZF3-ZPBP2* locus, we observed that risk for IBD, UC, CD, RA, PBC, asthma, and levels of HDL cholesterol increased with increasing levels of afucosylated agalactosylated and monogalactosylated glycans (IGP2 and IGP6) and decreased with increasing levels of fucosylation for all diseases, except IBD and CD. This suggests that a more proinflammatory and pro-ADCC glycan profile is associated with increased risk for these diseases. A similar pattern of increased agalactosylated glycan traits was observed in epidemiological studies of patients with IBD, CD, UC (*33*, *34*), and RA (*35*), and decreased fucosylation in UC, but not in CD (*34*).

In the same locus on chromosome 17, expression of *GSDMB* and *ORMDL3* in peripheral blood and B and T cells—and of *IKZF3* in peripheral blood—followed the opposite pattern of association with IgG glycans to that for the disease risks. While these genes are not glycosyltransferases and therefore do not have a direct influence on glycosylation levels, we showed a direct link between the *GSDMB-ORMDL3-IKZF3-ZPBP2* locus and *FUT8* and indirect links with *B4GALT1*, potentially explaining how pleiotropy with genes previously unrelated to glycosylation could influence glycosylation and suggesting why specific glycans are altered in these diseases.

One drawback of this study is the use of a relatively old HapMap2 imputation panel. However, at the onset of analyses, for many complex traits and expression studies, summary association statistics were only available for older panels. Still, using a newer imputation panel or even whole-genome sequencing data would increase the power of the study to detect rarer variants not well tagged in the HapMap2 imputation.

B cell development requires a regulatory network driven by coordinated activity of TFs, the end point being antibody production by plasma cells in response to antigen stimulus. The TFs IKZF1, IKZF3, RUNX1, and RUNX3 are lineage specific and have key roles in controlling the appropriate gene expression pattern at given time points, acting to both activate and repress gene expression and to alter the epigenetic landscape at functionally important genes. Furthermore, their relative expression levels are dynamically and reciprocally regulated (*19*). A limitation of our network is that it is one dimensional; that is, it cannot delineate hierarchal relationships nor indicate at which stage during B cell development these interactions occur. Functional characterization of individual SNPs and of these putative enhancers to establish causality is beyond the scope of this study and remains to be explored.

In conclusion, we have shown that GWAS together with gene prioritization and data-driven network analysis is a powerful strategy for identifying biologically meaningful regulatory mechanisms underlying a complex biological process as is IgG glycosylation. The complexity of our functional network appears to reflect both the complexity of glycans themselves and the complex differentiation process that B cells undergo to reach the terminally differentiated state of IgG-producing plasma cells. However, it is clear that variation in key TFs coupled with regulatory variation in glycosylation enzyme genes drives changes in IgG glycosylation, modifying IgG function and influencing health and disease.

## METHODS

All studies were approved by local research ethics committees, and all participants gave written informed consent. Details of participating cohorts, cohort-specific genotyping, quality control, and imputation performed before GWAS can be found in the Supplementary Note.

### Isolation of IgG and glycan analysis

Isolation of IgG and glycan analysis has been described in detail in previous studies. An example protocol and details about each study can be found in the Supplementary Note. Briefly, IgG was first isolated using affinity chromatography binding to protein G plates, followed by release and labeling of glycans with 2-AB (2-aminobenzamide) fluorescent dye. Glycans were then separated and quantified by hydrophilic interaction UPLC, resulting in 24 chromatographic peaks. Most of the peaks contain a single glycan structure, while some contain more than one, but with at least 63% of the peak being contributed by the most abundant glycan (*36*).

### Phenotype preprocessing

To reduce experimental variation in glycan measurements, before genetic studies, raw glycan data were total area normalized and batch corrected using the “ComBat” function of “sva” (*37*) R package centrally by the phenotype provider (Genos Ltd. for UPLC cohorts and Leiden University Medical Center for the Leiden Longevity Study). More detailed information on glycan preprocessing can be found in the Supplementary Note.

### Genetic association studies

#### Discovery

Discovery genome-wide association studies (GWAS) were performed in four cohorts of European descent, CROATIA-Korcula (*N* = 849), CROATIA-Vis (*N* = 802), ORCADES (Orkney Complex Disease Study) (*N* = 1960), and TwinsUK (N = 4479), with a combined sample size of 8090. Each glycan trait was first rank transformed to a normal distribution and then corrected for age, sex, cohort-specific covariates, and cryptic relatedness using linear mixed models and a kinship matrix estimated from genotyped data (as appropriate in each specific cohort). Within each cohort, a genome-wide association scan was performed based on the HapMap2 (release 22) imputed genetic data (or corresponding subset from a newer reference panel), assuming an additive linear model of association. Details of individual-level GWAS and parameters specific for each cohort can be seen in the Supplementary Note.

Cohort-level GWAS were examined for inconsistencies and informative data using the EasyQC software package (*38*), and results were pooled and analyzed with METAL (*39*) using a fixed-effect inverse-variance meta-analysis method. Before meta-analysis, each GWAS was corrected for genomic control inflation factor. The genomic control inflation factor varied from 0.95 to 1.05, suggesting little residual influence of population stratification. Additional genomic control was performed on the aggregated meta-analysis results. As suggested by Winkler *et al.* (*38*), GWAS cleaning and meta-analysis were performed by two independent analysts, and the obtained effect size estimates and *P* values of the two meta-analyses displayed perfect concordance.

Multiple testing was controlled for by considering an association as genome-wide significant if the *P* value of association was ≤2.4 × 10^−9^, as suggested by Li and Ji (*40*) and applied in Lauc *et al.* (*6*).

A locus was defined using the DEPICT tool (*10*) as a region spanned by SNPs in LD from the lead variant in the region. In cases where two such loci overlap, they were merged into a single locus.

#### Replication

Replication analysis was performed on 2368 samples from EGCUT (Estonian Genome Center, University of Tartu; *N* = 575), FINRISK (The National FINRISK Study; *N* = 552), COGS (Colorectal Cancer Genetics Susceptibility Study; *N* = 494), and SDRNT1BIO (Scottish Diabetes Research Network Type 1 Bioresource Study; *N* = 747). Top glycan–top SNP pairs from discovery meta-analysis from every genome-wide significant locus were tested for associations in each individual replication study and pooled using fixed-effect inverse-variance meta-analysis. The top SNP was defined as the SNP with the lowest *P* value in a locus. Replication significance level was set to *P* ≤ 1.9 × 10^−3^ (0.05 divided by the number of genome-wide significant loci, 27).

Additional validation was performed on glycosylation data measured with LCMS in the Leiden Longevity Study (*N* = 1842). Given that UPLC and LCMS measure similar but slightly different glycan traits (see “Validation genome-wide association studies” in the Supplementary Note), it was not possible to replicate the same discovery SNP-glycan pairs. Instead, we looked at associations of the top SNP from every locus with any LCMS glycan, with an aim of replicating a locus rather than SNP-glycan association. Therefore, we set the significance level to *P* ≤ 3.7 × 10^−5^ [0.05/50/27—Bonferroni correction for 50 directly measured LCMS glycan traits, as used in Wahl *et al.* (*8*), and 27 loci].

#### GWAS of gene expression

Peripheral blood eQTL summary-level genome-wide statistics were downloaded from the SMR Results Database (*41*) in August 2016. Briefly, this study meta-analyzed gene expression quantified with Illumina arrays in 5311 samples and HapMap2 reference panel imputed genotypes (*14*).

We also used eQTL summary-level genome-wide statistics for six circulating immune cell types [CD4^+^ T lymphocytes, CD8^+^ T lymphocytes, CD19^+^ B lymphocytes, CD14^+^ monocytes, CD15^+^ granulocytes, and platelets from the CEDAR dataset; (*13*)].

#### Conditional analyses and variance explained

To test for the association of secondary SNPs while conditioning on the top SNP in the region, we performed conditional analysis on summary statistics from the discovery meta-analysis. Briefly, this method performs forward stepwise selection on summary-level data, where, based on an LD estimated from a reference dataset in each iteration, the SNP with the strongest association in the region is added in the regression model until no additional SNPs reach genome-wide significance. GCTA stepwise variable selection (*9*) was performed using more than 6000 unrelated individuals from the Generation Scotland (*42*) as an independent reference sample and restricting collinearity to 0.9, with *P* ≤ 2.4 × 10^−9^ as a genome-wide significance level. Reported joint *P* value was corrected with genomic control inflation factor λ.

For every independent SNP*_i_*, we calculated the proportion of explained phenotypic variance as

$$\sigma_{i}=2*p_{i}*q_{i}*\beta_{i}^{2}$$

where β*_i_* is the effect estimate of SNP*_i_* in univariate meta-analysis. and *p_i_* and *q_i_* are the minor and major allele frequencies of SNP*_i_* calculated in the Generation Scotland cohort, respectively. For each glycan that has at least one significantly associated SNP, we calculated total univariate explained variance as a sum of the proportion of explained variance for all significant top SNPs for the given glycan.

To estimate the total variance explained by all independently contributing SNPs (total joint variance), we used the following procedure. For each glycan, we estimated the total joint variance by summing the contribution of each independently contributing SNP defined as

$$\sigma_{i}^{J}=2*p_{i}*q_{i}*\beta_{i}^{u}*\beta_{i}^{J}$$

where $\beta_{i}^{u}$ is the effect estimate of SNP*_i_* in the univariate analysis, and $\beta_{i}^{J}$ is the joint effect estimate of the same SNP in the joint analysis.

### Prioritizing genes associated with IgG N-glycosylation

For the genome-wide suggestive and significant loci, we explored potential causative genes at each locus using a strategy that combined publicly available datasets and prioritization tools and eQTLs from peripheral blood– and immune cell type–specific datasets.

#### Coding variation

To obtain the putative functional effect of associated variants, significantly or suggestively associated SNPs were annotated with VEP (*11*) in May 2016. Because of alternative splicing, each gene can have more than one transcript, and, consequently, depending on the transcript, one SNP can have a different effect on protein function.

#### SMR/HEIDI analysis for pleiotropy with gene expression

To test for potential pleiotropy between gene expression and IgG glycosylation, we performed SMR with HEIDI analysis, developed by Zhu *et al.* (*12*). SMR analysis provides evidence for pleiotropy but cannot distinguish if the associations are driven by the same or highly correlated but distinct causal variants. The subsequent HEIDI test allowed us to distinguish pleiotropy from LD. The test was performed on a publicly available peripheral blood dataset from Westra *et al* (*14*) and immune cell–specific gene expression from the CEDAR dataset (*13*). Among others, this dataset contains expression in B lymphocytes (CD19), helper T lymphocytes (CD4), cytotoxic T cells (CD8), macrophages (CD14), neutrophils (CD15), and platelets (PLA).

We set a threshold for the SMR test at *P*_SMR_ ≤ 1.9 × 10^−5^, corresponding to a Bonferroni correction for 2622 tests, number of regions where genome-wide significant top regional IgG glycan SNP (or its proxy) was also available in any of the gene expression associations. All regions with significant *P*_SMR_ and *P*_HEIDI_ ≥ 0.05 were considered to exhibit concordance in regional association patterns and therefore showed evidence of sharing the underlying unobserved causal variant. For these regions, we can suggest that they are pleiotropic. Details of the algorithm can be seen in the Supplementary Note.

#### Prioritization using DEPICT

DEPICT (*10*) was run on the merged list of independent SNPs obtained from the GCTA-COJO analysis to identify gene sets enriched for genes near associated variants. Suggestive independent SNPs (*P* ≤ 5 × 10^−8^) were submitted to DEPICT, release 194 (*10*). The list of independent SNPs was created by merging glycan-wise GCTA-COJO results, resulting in 113 SNPs. Given that different glycans can have a different lead SNP (but in high LD), this list was additionally pruned by applying PLINK clumping (*43*), where all SNPs within 500 kb and LD *R*^2^ > 0.1 of the SNP with the strongest association were assigned to the same clump. LD was estimated using 1000 Genomes Project Phase 1 CEU [Utah Residents (CEPH) with Northern and Western European Ancestry], GBR (British in England and Scotland), and TSI (Toscani in Italy) data.

#### Gene set enrichment analysis

GO enrichment analysis was performed using FUMA GENE2FUNC (*15*) analysis based on MSigDB c5 with default parameters and *All genes* as background genes. Glycosylation-related gene sets were defined as any GO gene set whose description contained words “glyc,” “sacch. fucose,” “carbo,” or “hexose.” Immune system–related gene sets were defined in the same manner, but searching for words “immune,” “B_CELL,” “lymphocyte,” “leukocyte,” “T_CELL,” “hemopoi,” and “myeloid,” while transcription-related gene-sets were defined using the keywords “transcription” or “expression.” DEPICT pathways and tissue enrichments analyses were performed as described above.

### Functional network of genes associated with IgG N-glycosylation

To suggest a functional relationship of loci associated with IgG glycosylation, we performed pairwise association analyses of glycome-wide effects of lead SNPs. Glycome-wide effect of the SNP was defined as the vector of *z* scores [*z* score = β/*se*(β)] of this SNP on each of glycans.

Before analysis, we removed 15 derived traits (IGP41 to IGP54) that represent directly measured glycans (IGP1 to IGP15) that were normalized with the surface area of neutrally charged glycans (see the Supplementary Note for details).

We then constructed glycome-wide effects for all 27 lead SNPs from the meta-analysis results for 62 glycans, resulting in 27 vectors of 62 *z* scores. For every pair of SNPs, we computed the Spearman’s correlation coefficient and corresponding *P* value between 27 glycome-wide effects.

The network of significant Spearman’s correlations [*P* value corrected for (27 × 26)/2 = 351 tests, *P* ≤ 1.4 × 10^−4^] was visualized using Cytoscape (*44*), where each node represents one lead SNP annotated with the gene prioritized in the region of that SNP and width and color intensity of edges present squared Spearman’s correlation coefficient of the two nodes. We next performed clustering analysis of the full correlation matrix by applying hierarchical clustering with Euclidean distance and complete linkage. To validate the network, we performed permutation analysis and compared the network with STRING PPI networks (*16*).

The permutation analysis was performed by estimating the correlation of glycome-wide effects of every top SNP and glycome-wide effects of 100,000 random SNPs. To ensure that no glycosylation-associated SNPs were included in the permutation analysis, the following procedure was used. From the list of all HapMap release 22 SNPs (2,574,585 SNPs), all variants from the associated loci were removed, resulting in 2,510,568 remaining SNPs. An additional 6641 SNPs were removed because they had *P*_GWAS_ ≤ 5 × 10^−5^ in at least one glycosylation GWAS. The final list contained 2,503,927 SNPs. From this list, 100,000 SNPs were randomly selected for the permutation analysis. A Spearman’s correlation between glycome-wide effects of each of the 27 top SNPs with glycome-wide effects of 100,000 random SNPs was computed. These correlations were then compared with correlations used to construct the network.

Additional validation was performed by comparing the network obtained by submitting IgG N-glycosylation candidate genes to the STRING database of PPIs (version 10.5, accessed in September 2017) (*16*).

ChIP-seq data (.narrowPeak files) for the TFs were downloaded from http://hgdownload.cse.ucsc.edu/goldenPath/hg19/encodeDCC/ for the GM12878 LCL. The peak-motifs tool (*18*) in the Regulatory Sequence Analysis Tools was used for motif discovery in the peak sequences of the ChIP-seq datasets. To assess whether glycosylation-associated SNPs disrupt or introduce TF-binding sites, we first filtered out all SNPs with no predicted effect on TF binding, with TF weight score *P* values higher than 1.8 × 10^−8^ (0.05/2,764,712, Bonferroni corrected for the number of tests performed). We then further filtered out the SNPs whose alternate alleles had similar TF-binding scores and focused on SNPs for which one allele results in loss of TF-binding site. To compare frequencies of TF-binding site disruptions of glycosylation SNPs and other SNPs in the region, we performed the same analysis using all significantly and suggestively associated glycosylation SNPs (associated SNPs) and all SNPs within 50 kb of every glycosylation locus whose association *P* value was ≤5 × 10^−4^ (nonassociated SNPs) and compared frequency of SNPs that are predicted to significantly (Bonferroni-corrected *P* ≤ 1.8 × 10^−8^) disrupt binding for the given TF.

Hi-C data derived from GM12878, digested with Mbo I, were interrogated using the online tool available at http://higlass.io/app/ (*45*). More details can be found in the Supplementary Note.

### In vitro validation of the functional links between *IKZF1* and *FUT8*

#### ShRNA cloning, cell culture, transfections, and shRNA knockdown

Four shRNAs targeting the coding region or the 3′ untranslated region of all *IKZF1* isoforms were designed using Block-iT RNAi Designer (https://rnaidesigner.thermofisher.com/rnaiexpress/). Single-stranded oligonucleotides for IKZF1 shRNA and a sequence with no corresponding target in the human genome were annealed and cloned into pENTR/H1/TO vector and used for electroporation of an IgG1-secreting human LCL, MATAT6. RNA was extracted to assess gene knockdown efficiency for each shRNA tested by quantitative PCR (qPCR), and the IKZF1 shRNA resulting in the most significant knockdown was used for subsequent experiments. More details can be found in the Supplementary Note.

#### RNA, complementary DNA, and real-time PCR

RNA was extracted from MATAT6 cells and stable shRNA lines, followed by on-column DNase (deoxyribonuclease) digestion and synthesis of complementary DNA and qPCR. qPCR gene expression assays were performed for *IKZF1*, *FUT8*, *IKZF3*, and *HPRT1*. Samples were run in triplicate. Relative gene expression level was determined using the comparative *C*_t_ (cycle threshold) method. Statistically significant differences in gene expression were determined using the paired *t* test. More details can be found in the Supplementary Note.

#### Western blot analysis

Whole-cell lysates were prepared by lysing cells in radioimmunoprecipitation assay buffer on ice. Jurkat nuclear extract and human embryonic kidney 293T lysates were used as positive and negative controls for IKZF1. Ten micrograms of total protein was reduced and denatured before separation on 4 to 12% Bis-Tris NuPAGE gel. Membranes were probed using rabbit polyclonal anti-Ikaros and mouse anti–α-tubulin antibodies. Secondary antibodies were horseradish peroxidase–conjugated anti-rabbit IgG or anti-mouse IgG. Antibody detection was achieved using the enhanced chemiluminescence detection system. More details can be found in the Supplementary Note.

#### Glycan profiling of secreted IgG

Five million cells from stable bulk cultures of shRNA-expressing MATAT6 cells were washed in phosphate-buffered saline and then resuspended in (serum-free) Opti-MEM. After 72 hours, conditioned media were collected by centrifugation and immediately frozen at −80°C. The glycan profile of secreted IgG in samples collected on at least two occasions from IKZF1 shRNA lines (*n* = 3) or random shRNA lines (*n* = 2) was determined by LCMS as described under the validation LCMS study in the Supplementary Note. A paired *t* test (GraphPad QuickCalcs) was performed to assess significant differences in glycosylation features.

#### Chromatin immunoprecipitation

ChIP assays were performed using ExactaChIP buffers (R&D Systems), as described by the manufacturer, except for the modifications described in the Supplementary Note. ChIP input was incubated with either anti-IKZF1 or goat IgG isotype control antibody overnight at 4°C with rotation. Five micrograms of biotinylated anti-goat IgG was added for a further 2 hours before the addition of streptavidin-agarose beads. Agarose beads were collected by centrifugation. After the final wash, Chelating resin solution was added to the beads, and the samples were boiled for 10 min. ChIP-PCR was performed using primers flanking a binding site upstream of *FUT8* identified by ChIP-seq analysis in the GM12878 cell line to confirm that IKZF1-DNA complexes also occur in MATAT6 cells. PCR products were analyzed on 2% (w/v) agarose gel by electrophoresis in tris-borate EDTA buffer.

#### SMR/HEIDI analysis for pleiotropy with complex traits

To assess regional association concordance between IgG glycosylation and other complex traits (SMR/HEIDI test), we were able to download summary-level statistics including signed regression coefficient estimates and SE of these estimates for 10 traits (see the Supplementary Note for details). For each unique locus and glycan-trait combination for the test, we used only the SNP with the lowest *P* value in glycan GWAS. For all the available traits, the same procedure as outlined in the “SMR/HEIDI analysis for pleiotropy with gene expression” was applied.

## Supplementary Material

http://advances.sciencemag.org/cgi/content/full/6/8/eaax0301/DC1

Download PDF

Table S1

Glycosylation of immunoglobulin G is regulated by a large network of genes pleiotropic with inflammatory diseases

## SUPPLEMENTARY MATERIALS

Supplementary material for this article is available at http://advances.sciencemag.org/cgi/content/full/6/8/eaax0301/DC1 Supplementary Note Appendix Table 1. Participating studies. Appendix Table 2. Genotyping overview. Appendix Table 3. Overview of imputation software and reference panels. Appendix Table 4. Description of structures for UPLC IgG glycans. Appendix Table 5. Details of genome-wide association analyses. Appendix Table 6. Individual GWAS file-level quality control. Appendix Table 7. Description of structures for LCMS glycans. Appendix Table 8. Summary-level statistics downloaded for SMR or HEIDI test. Appendix Table 9. DEPICT gene prioritization. Appendix Table 10. SNPs associated with IgG glycosylation with nonsynonymous amino acid change. Appendix Table 11. Strongest eQTL for each probe in each cell type in the CEDAR dataset. Appendix Figure 1. Phenotypic correlation of UPLC IgG *N*-glycans. Fig. S1. Glycome-wide effect estimates of genome-wide significant loci. Fig. S2A. RUNX3 binding in the proximity of MGAT3 rs8137426, SNP strongly associated with IgG N-glycosylation is the region bound by RUNX3, in the proximity of MGAT3. Fig. S2B. Top SNPs in *FUT8* locus lie in the same chromosomal topological associating domain as the transcription start site of FUT8. Table S1. Comparison of *P* values from current meta-analysis (*P*-new) and Lauc *et al*. (*P*-old), Shen *et al.* (*P*-multivariate), and Wahl *et al*. (*P*-LCMS). Table S2. Results of replication and validation analysis. Table S3. Phenotypic variance explained by significantly associated SNPs (*P* ≤ 2.4 × 10^−9^). Table S4. FUMA GO gene set enrichment analysis. Table S5. DEPICT analysis of gene set enrichment. Table S6. Correlation of glycome-wide effects of top genome-wide significant SNPs associated with IgG glycosylation. Table S7. STRING PPI analysis of genes prioritized in loci associated with IgG N-glycosylation. Table S8. TF motif alterations by glycosylation-associated SNPs. Table S9. Effect of IgG glycosylation–associated SNPs on TF motif–binding disruption compared with nonassociated SNPs from the region. Table S10. Degree of fucosylation of IgG secreted from MATAT6 cells. References (*46*–*83*)

## Appendix

View/request a protocol for this paper from *Bio-protocol*.
