# Supplementary material for: Glycosylation of immunoglobulin G is regulated by a large network of genes pleiotropic with inflammatory diseases
Source: Sci Adv. 2020 Feb 19;6(8):eaax0301. doi: 10.1126/sciadv.aax0301 (PMC7030929; doi:10.1126/sciadv.aax0301)
Supplement: http://advances.sciencemag.org/cgi/content/full/6/8/eaax0301/DC1 [file supp_6_8_eaax0301__index.html]

Science Advances | Science AdvancesAAASSearchScience AdvancesMenu

## Supplementary Materials

**The PDF file includes:**

- Supplementary Note
- Appendix Table 1. Participating studies.
- Appendix Table 2. Genotyping overview.
- Appendix Table 3. Overview of imputation software and reference panels.
- Appendix Table 4. Description of structures for UPLC IgG glycans.
- Appendix Table 5. Details of genome-wide association analyses.
- Appendix Table 6. Individual GWAS file-level quality control.
- Appendix Table 7. Description of structures for LCMS glycans.
- Appendix Table 8. Summary-level statistics downloaded for SMR or HEIDI test.
- Appendix Table 9. DEPICT gene prioritization.
- Appendix Table 10. SNPs associated with IgG glycosylation with nonsynonymous amino acid change.
- Appendix Table 11. Strongest eQTL for each probe in each cell type in the CEDAR dataset.
- Appendix Figure 1. Phenotypic correlation of UPLC IgG *N*-glycans.
- Fig. S1. Glycome-wide effect estimates of genome-wide significant loci.
- Fig. S2A. RUNX3 binding in the proximity of MGAT3 rs8137426, SNP strongly associated with IgG N-glycosylation is the region bound by RUNX3, in the proximity of MGAT3.
- Fig. S2B. Top SNPs in *FUT8* locus lie in the same chromosomal topological associating domain as the transcription start site of FUT8.
- References (*46*–*83*)

Download PDF

**Other Supplementary Material for this manuscript includes the following:**

- Table S1 (Microsoft Excel format). Comparison of *P* values from current meta-analysis (*P*-new) and Lauc *et al*. (*P*-old), Shen *et al.* (*P*-multivariate), and Wahl *et al*. (*P*-LCMS).
- Table S2 (Microsoft Excel format). Results of replication and validation analysis.
- Table S3 (Microsoft Excel format). Phenotypic variance explained by significantly associated SNPs (*P* ≤ 2.4 × 10^−9^).
- Table S4 (Microsoft Excel format). FUMA GO gene set enrichment analysis.
- Table S5 (Microsoft Excel format). DEPICT analysis of gene set enrichment.
- Table S6 (Microsoft Excel format). Correlation of glycome-wide effects of top genome-wide significant SNPs associated with IgG glycosylation.
- Table S7 (Microsoft Excel format). STRING PPI analysis of genes prioritized in loci associated with IgG N-glycosylation.
- Table S8 (Microsoft Excel format). TF motif alterations by glycosylation-associated SNPs.
- Table S9 (Microsoft Excel format). Effect of IgG glycosylation–associated SNPs on TF motif–binding disruption compared with nonassociated SNPs from the region.
- Table S10 (Microsoft Excel format). Degree of fucosylation of IgG secreted from MATAT6 cells.

**Files in this Data Supplement:**

- Adobe PDF - aax0301\_SM.pdf
