# Supplementary material for: Glycosylation of immunoglobulin G is regulated by a large network of genes pleiotropic with inflammatory diseases
Source: Sci Adv. 2020 Feb 19;6(8):eaax0301. doi: 10.1126/sciadv.aax0301 (PMC7030929; doi:10.1126/sciadv.aax0301)
Supplement: Download PDF [file aax0301_SM.pdf]

Science Advances
AAAS

advances.sciencemag.org/cgi/content/full/6/8/eaax0301/DC1

Supplementary Materials for

Glycosylation of immunoglobulin G is regulated by a large network of genes pleiotropic with inflammatory diseases

Lucija Klarić*, Yakov A. Tsepilov, Chloe M. Stanton, Massimo Mangino, Timo Tõnis Sikka, Tõnu Esko, Eugene Pakhomov, Perttu Salo, Joris Deelen, Stuart J. McGurnaghan, Toma Keser, Frano Vučković, Ivo Ugrina, Jasminka Krištić, Ivan Gudelj, Jerko Štambuk, Rosina Plomp, Maja Pučić-Baković, Tamara Pavić, Marija Vilaj, Irena Trbojević-Akmačić, Camilla Drake, Paula Dobrinić, Jelena Mlinarec, Barbara Jelušić, Anne Richmond, Maria Timofeeva, Alexander K. Grishchenko, Julia Dmitrieva, Mairead L. Bermingham, Sodbo Zh. Sharapov, Susan M. Farrington, Evropi Theodoratou, Hae-Won Uh, Marian Beekman, Eline P. Slagboom, Edouard Louis, Michel Georges, Manfred Wuhrer, Helen M. Colhoun, Malcolm G. Dunlop, Markus Perola, Krista Fischer, Ozren Polasek, Harry Campbell, Igor Rudan, James F. Wilson, Vlatka Zoldoš, Veronique Vitart, Tim Spector, Yurii S. Aulchenko, Gordan Lauc, Caroline Hayward

*Corresponding author. Email: lklaric@genos.hr

Published 19 February 2020, *Sci. Adv.* **6**, eaax0301 (2020)
DOI: 10.1126/sciadv.aax0301

The PDF file includes:

Supplementary Note
Appendix Table 1. Participating studies.
Appendix Table 2. Genotyping overview.
Appendix Table 3. Overview of imputation software and reference panels.
Appendix Table 4. Description of structures for UPLC IgG glycans.
Appendix Table 5. Details of genome-wide association analyses.
Appendix Table 6. Individual GWAS file-level quality control.
Appendix Table 7. Description of structures for LCMS glycans.
Appendix Table 8. Summary-level statistics downloaded for SMR or HEIDI test.
Appendix Table 9. DEPICT gene prioritization.
Appendix Table 10. SNPs associated with IgG glycosylation with nonsynonymous amino acid change.
Appendix Table 11. Strongest eQTL for each probe in each cell type in the CEDAR dataset.
Appendix Figure 1. Phenotypic correlation of UPLC IgG *N*-glycans.
Fig. S1. Glycome-wide effect estimates of genome-wide significant loci.
Fig. S2A. RUNX3 binding in the proximity of MGAT3 rs8137426, SNP strongly associated with IgG N-glycosylation is the region bound by RUNX3, in the proximity of MGAT3.
Fig. S2B. Top SNPs in *FUT8* locus lie in the same chromosomal topological associating domain as the transcription start site of FUT8.
References (*46*–*83*)

Other Supplementary Material for this manuscript includes the following:

(available at advances.sciencemag.org/cgi/content/full/6/8/eaax0301/DC1)

Table S1 (Microsoft Excel format). Comparison of *P* values from current meta-analysis (*P*-new) and Lauc *et al*. (*P*-old), Shen *et al.* (*P*-multivariate), and Wahl *et al*. (*P*-LCMS).
Table S2 (Microsoft Excel format). Results of replication and validation analysis.
Table S3 (Microsoft Excel format). Phenotypic variance explained by significantly associated SNPs ($P \leq 2.4 \times 10^{-9}$).
Table S4 (Microsoft Excel format). FUMA GO gene set enrichment analysis.
Table S5 (Microsoft Excel format). DEPICT analysis of gene set enrichment.
Table S6 (Microsoft Excel format). Correlation of glycome-wide effects of top genome-wide significant SNPs associated with IgG glycosylation.
Table S7 (Microsoft Excel format). STRING PPI analysis of genes prioritized in loci associated with IgG N-glycosylation.
Table S8 (Microsoft Excel format). TF motif alterations by glycosylation-associated SNPs.
Table S9 (Microsoft Excel format). Effect of IgG glycosylation–associated SNPs on TF motif–binding disruption compared with nonassociated SNPs from the region.
Table S10 (Microsoft Excel format). Degree of fucosylation of IgG secreted from MATAT6 cells.

Supplementary Note

Methods

Descriptions of the glycan data can be found in Huffman *et al*(*46*).

Isolation of IgG and glycan analysis

Isolation of IgG and glycan analysis has been described in a greater detail in the following studies: CROATIA-Korcula and CROATIA-Vis(*36*), ORCADES(*47*), TWINSUK(*48*), COGS, EGCUT, FINRISK(*49*). Details of the liquid chromatography electrospray mass spectrometry used to quantify Fc bound IgG glycopeptides can be found in Plomp *et al*(*46*).

IgG isolation. IgG was isolated from blood plasma samples (70-80 uL) by affinity chromatography binding to CIM® Protein G 96-well plate (BIA Separations, Ajdovščina, Slovenia), using vacuum manifold (Millipore Corporation, Billerica, MA, USA). All steps during the isolation procedure were performed at around 380 mm Hg, except plasma sample application and IgG elution steps (around 200 mm Hg). Protein G plate was washed with 2 mL ultra-pure water (18 MΩ cm at 25 °C), 2 mL 1x PBS pH 7.4, 1 mL 0.1 mol L-1 formic acid; 2 mL 10x PBS; and equilibrated with 4 mL 1x PBS pH 7.4.

Plasma samples, ψ(sample, 1x PBS pH 7.4) = 1:7, were applied to the protein G plate. Unbound proteins were washed away with 3x 2 mL 1x PBS pH 7.4. Bound IgG was eluted with 1 mL 0.1 mol L-1 formic acid and neutralized with 1 mol L-1 ammonium hydrogencarbonate to pH 7.0.

Protein G plate was then washed with 1 mL 0.1 mol L-1 formic acid, 2 mL 10x PBS, 4 mL 1x PBS pH 7.4, 2 mL storage buffer (ethanol φ = 20 %, 20 mmol L-1 Tris, 0.1 mol L-1 NaCl, titrated with HCl to pH 7.4), and plate stored at 4 °C.

N-glycan release and labelling. The whole procedure was done in a 96-well plate manner and ultra-pure water was used throughout. Dried IgG samples (150-200 ug) were denatured with the addition of 30 μl SDS (γ = 13.3 g L-1) and by incubation at 65 °C for 10 min. After cooling down to room temperature for 30 min, 10 μl of Igepal-CA630 (φ = 4 %) was added and mixture was shaken for 15 min on a plate shaker. N-glycans were released with the addition of 1.2 U of PNGase F (Promega, Madison, WI, USA) in 10 μL 5x PBS and incubation at 37 °C for 18 hours. Released N-glycans were labeled with 2-AB. The labeling mixture was freshly prepared by dissolving 2-AB (final γ = 19.2 mg mL-1) and 2-PB (final γ = 44.8 mg mL-1) in the mixture of DMSO and glacial acetic acid, ψ(DMSO, CH3COOH) = 7:3. To each N-glycan sample in the 96-well plate 25 μl of labeling mixture was added and the plate was sealed using adhesive seal. Mixing was achieved by shaking for 10 min, followed by 2 hour incubation at 65 °C.

Clean-up of 2-AB labeled glycans. Free fluorescent label, excess of reagents and proteins were removed from the samples using hydrophilic interaction liquid chromatography solid phase extraction (HILIC-SPE). After cooling down to room temperature for 30 min, 700 μL of acetonitrile (previously cooled down to 4 °C) was added to each sample (total volume of 775 μl). Clean-up procedure was performed on hydrophilic 0.2 μm AcroPrep GHP filter plate. Solvent was removed by application of vacuum using a vacuum manifold at around 25 mm Hg. All wells were prewashed with 200 μL ethanol in water (φ = 70 %), 200 μL ultra-pure water and 200 μL acetonitrile in water (φ = 96 %, previously cooled down to 4 °C). The samples were loaded to the wells, and after short incubation subsequently washed 5x 200 μL acetonitrile in water (φ = 96 %, previously cooled down to 4 °C). Glycans were eluted with 2x 90 μL of ultra-pure water after 15 min shaking at room temperature and combined eluates were stored at -20 °C until ultra performance liquid chromatography (UPLC) analysis.

Glycan analysis by ultra performance liquid chromatography. Fluorescently labeled N-glycans were separated by HILIC on an Acquity UPLC instrument (Waters, Milford, MA, USA) consisting of a quaternary solvent manager, sample manager and a FLR fluorescence detector set with excitation and emission wavelengths of 250 and 428 nm, respectively. The instrument was under the control of Empower 3 software, build 3471 (Waters). Labeled N-glycans were separated on a Waters BEH Glycan chromatography column, 100 × 2.1 mm i.d., 1.7 μm BEH particles, with 100 mmol L-1 ammonium formate, pH 4.4, as solvent A and acetonitrile as solvent B. The samples were prepared in acetonitrile, ψ(sample, acetonitrile) = 20:80. The separation method used a linear gradient of 75% to 62% acetonitrile (vol/vol) at flow rate of 0.4 mL/min in a 25-minute analytical run. Samples were maintained at 10 °C before injection, and the separation temperature was 60 °C. The system was calibrated using an external standard of hydrolyzed and 2-AB labeled glucose oligomers from which the retention times for the individual glycans were converted to glucose units. Data processing was performed using an automatic processing method with a traditional integration algorithm after which each chromatogram was manually corrected to maintain the same intervals of integration for all the samples. The chromatograms were all separated in the same manner into 24 peaks and the amount of glycans in each peak was expressed as percentage of total integrated area (% Area) (*36*).

Participating studies and genetic data pre-processing

All genotyping, quality control, imputation and genome-wide association studies were performed in the respective research centres. Details of participating studies can be seen in Appendix Table 1 and details of genetic analyses can be seen in Appendix Tables 2, 3, and 4.

Appendix Table 1. Participating studies.

Cohort name	Full name of the study	Study design	Technology	# Analysed	Median Age (range)	F	M
TwinsUK (*50, 51*)	UK Adult Twin Register	Population based	UPLC	4479	54 (17-83)	4937	0
ORCADES (*52*)	The Orkney Complex Disease Study	Population Based	UPLC	1960	54 (16-100)	1236	794
CROATIA-Korcula (*53*)	10001 Dalmatians	Population Based	UPLC	849	57 (18-98)	595	320
CROATIA-Vis (*54*)	10001 Dalmatians	Population Based	UPLC	802	57 (18-93)	521	369
EGCUT (*55*)	Estonian Genome Center, University of Tartu	Population Based, selected for developing IBD, RA, CRC	UPLC	575	71 (31-88)	290	285
FINRISK (*56*)	The National FINRISK Study	Disease susceptibility	UPLC	552	53 (24-74)	400	392
COGS (*57, 58*)	Colorectal Cancer Genetics Susceptibility Study	Cancer free population based controls	UPLC	494	53 (21-74)	244	284
SDRNT1BIO (*59*)	Scottish Diabetes Research Network Type 1 Bioresource Study	Patient cohort	UPLC	767	43 (16-94)	401	417
LLS (*60*)	Leiden Longevity Study	Family based	LC-ESI-MS	1841	59 (30-79)	974	867

Appendix Table 2. Genotyping overview.

Cohort	Platform	ID call rate	MAF	SNP call rate	HWE P	#SNPs that met QC
TwinsUK	Illumina HumanHap300 and Illumina HumanHap610Q	>95%	0.01	>98%	>1e-06	553487
ORCADES	HumanHap300v2 and OmniExpress	>97%	0.01	>98%	>10e-10	741151
CROATIA-Korcula	Illumina HumanHap 370CNV DUO/QUAD Phase 1	>97%	0.01	>98%	>10e-06	316730
CROATIA-Vis	Illumina HumanHap300v1	>95%	0.01	>98%	>10e-06	308996
EGCUT	Illumina OmniExpress	>95%	0.01	>95%	>10e-6	691978
FINRISK	Illumina HumanHap 610-Quad	>95%	0.01	>95%	>10e-6	485919
COGS	Illumina HumanHap300 and HumanHap240S	>95%	0.01	>95%	0.00001	514177
SDRNT1BIO	Illumina OmniExpress	>95%	0.01	>95%	>1e-06	690798
Generation Scotland	Illumina HumanOmniPlusExome	>98%	0.01	>97%	>1e-06	706980
Leiden Longevity Study	Illumina660 W; Illumina OmniExpress	>95%	0.01	>95%	> 1e-04	296619

ID call rate - percentage of SNPs successfully genotyped in every individual; SNP call rate - percentage of samples in which the given genotype was successfully called; HWE - Hardy-Weinberg Equilibrium; MAF - minor allele frequency

Appendix Table 3. Overview of imputation software and reference panels.

Cohort	Imputation software	Reference panel
TwinsUK	IMPUTE2	1000 Genomes
ORCADES	SHAPEIT/Impute2	1000 Genomes
CROATIA-Korcula	MACH version 1.15	HapMap2 release 22
CROATIA-Vis	MACH version 1.15	HapMap2 release 22
EGCUT	IMPUTE2	HapMap2 release 22
FINRISK	SHAPEIT v1/ IMPUTE v2.2.2	1000 Genomes
COGS	SHAPEIT/Impute2	1000 Genomes, phase 1 Integrated haplotypes, released June 2014
SDRNT1BIO	ShapeIt2/IMPUTE2	1000 Genomes
Generation Scotland	ShapeIt2/IMPUTE2	1000 Genomes
Leiden Longevity Study	IMPUTE2	HapMap2 release 22

Glycan data pre-processing

Each of the 24 raw glycan intensities was divided by the total chromatographic area of the sample, followed by log transformation and exclusion of extreme values that most likely present technical outliers. Batch correction was performed with an empirical Bayes method (*61*), as implemented in the “ComBat” function of “sva” (*37*) R package. Derived traits, traits combining glycans with similar structural and chemical properties (for example – fucosylation – sum of all glycans that have core fucose) were then computed on exponentiated measured glycans, using the “iudt” and “ildt” functions from the glycanr package (*62*), resulting in a final list of 77 glycan traits(*36*). The list of directly measured and derived glycan traits, together with formulas used for their calculation can be seen in the Appendix Table 4. The same procedure, with the difference of normalising glycopeptide data within subclass rather than on the whole dataset was applied to LCMS glycan data.

Appendix Table 4. Description of structures for UPLC IgG glycans.

Adapted from Pucic *et al*(*36*)

GWAS Name	Glycan trait/Formula	DESCRIPTION
**Measured glycan traits**		
IGP1	GP1	The percentage of FA1 glycan in total IgG glycans
IGP2	GP2	The percentage of G0 glycan in total IgG glycans
IGP3	GP4	The percentage of G0F glycan in total IgG glycans
IGP4	GP5	The percentage of M5 glycan in total IgG glycans
IGP5	GP6	The percentage of G0FN glycan in total IgG glycans
IGP6	GP7	The percentage of G1 glycan in total IgG glycans
IGP7	GP8	The percentage of G1F[6] glycan in total IgG glycans
IGP8	GP9	The percentage of G1[3]F glycan in total IgG glycans
IGP9	GP10	The percentage of G1[6]FN glycan in total IgG glycans
IGP10	GP11	The percentage of G1[3]FN glycan in total IgG glycans
IGP11	GP12	The percentage of G2 glycan in total IgG glycans
IGP12	GP13	The percentage of G2N glycan in total IgG glycans
IGP13	GP14	The percentage of G2F glycan in total IgG glycans
IGP14	GP15	The percentage of G2FN glycan in total IgG glycans
IGP15	GP16	The percentage of G1FS1 glycan in total IgG glycans
IGP16	GP17	The percentage of G2S1 glycan in total IgG glycans
IGP17	GP18	The percentage of G2FS1 glycan in total IgG glycans
IGP18	GP19	The percentage of G2FNS1 glycan in total IgG glycans
IGP19	GP20	Structure not determined
IGP20	GP21	The percentage of G2S2 glycan in total IgG glycans
IGP21	GP22	The percentage of G2NS2 glycan in total IgG glycans
IGP22	GP23	The percentage of G2FS2 glycan in total IgG glycans
IGP23	GP24	The percentage of G2FNS2 glycan in total IgG glycans
**Derived glycan traits**		
IGP24	SUM(GP16 + GP18 + GP23) / SUM(GP16 + GP18 + GP23 + GP8 + GP9 + GP14) * 100	The percentage of sialylation of fucosylated galactosylated structures without bisecting GlcNAc in total IgG glycans
IGP25	SUM(GP19 + GP24) / SUM(GP19 + GP24 + GP10 + GP11 + GP15) * 100	The percentage of sialylation of fucosylated galactosylated structures with bisecting GlcNAc in total IgG glycans
IGP26	SUM(GP16 + GP18 + GP23) / SUM(GP16 + GP18 + GP23 + GP4 + GP8 + GP9 + GP14) * 100	The percentage of sialylation of all fucosylated structures without bisecting GlcNAc in total IgG glycans
IGP27	SUM(GP19 + GP24) / SUM(GP19 + GP24 + GP6 + GP10 + GP11 + GP15) * 100	The percentage of sialylation of all fucosylated structures with bisecting GlcNAc in total IgG glycans
IGP28	GP16 / SUM(GP16 + GP8 + GP9) * 100	The percentage of monosialylation of fucosylated monogalactosylated structures without bisecting GlcNAc in total IgG glycans
IGP29	GP18 / SUM(GP18 + GP14 + GP23)	The percentage of monosialylation of fucosylated

	* 100	digalactosylated structures without bisecting GlcNAc in total IgG glycans
IGP30	GP23 / SUM(GP23 + GP14 + GP18) * 100	The percentage of disialylation of fucosylated digalactosylated structures without bisecting GlcNAc in total IgG glycans
IGP31	GP19 / SUM(GP19 + GP15 + GP24) * 100	The percentage of monosialylation of fucosylated digalactosylated structures with bisecting GlcNAc in total IgG glycans
IGP32	GP24 / SUM(GP24 + GP15 + GP19) * 100	The percentage of disialylation of fucosylated digalactosylated structures with bisecting GlcNAc in total IgG glycans
IGP33	SUM(GP16 + GP18 + GP19) / SUM(GP23 + GP24)	Ratio of all fucosylated monosialylated and disialylated structures (+/- bisecting GlcNAc) in total IgG glycans
IGP34	SUM(GP16 + GP18) / GP23	Ratio of fucosylated monosialylated and disialylated structures (without bisecting GlcNAc) in total IgG glycans
IGP35	GP19 / GP24	Ratio of fucosylated monosialylated and disialylated structures (with bisecting GlcNAc) in total IgG glycans
IGP36	SUM(GP19 + GP24) / SUM(GP16 + GP18 + GP23)	Ratio of all fucosylated sialylated structures with and without bisecting GlcNAc in total IgG glycans
IGP37	GP19 / SUM(GP16 + GP18)	Ratio of fucosylated monosialylated structures with and without bisecting GlcNAc in total IgG glycans
IGP38	GP19 / SUM(GP16 + GP18 + GP19)	The incidence of bisecting GlcNAc in all fucosylated monosialylated structures in total IgG glycans in total IgG glycans
IGP39	GP24 / GP23	Ratio of fucosylated disialylated structures with and without bisecting GlcNAc in total IgG glycans
IGP40	GP24 / SUM(GP23 + GP24)	The incidence of bisecting GlcNAc in all fucosylated disialylated structures in total IgG glycans
IGP41	GP1 / GPn* 100	The percentage of FA1 glycan in total neutral IgG glycans (GPn)
IGP42	GP2 / GPn* 100	The percentage of G0 glycan in total neutral IgG glycans (GPn)
IGP43	GP4 / GPn* 100	The percentage of G0F glycan in total neutral IgG glycans (GPn)
IGP44	GP5 / GPn* 100	The percentage of M5 glycan in total neutral IgG glycans (GPn)
IGP45	GP6 / GPn* 100	The percentage of G0FN glycan in total neutral IgG glycans (GPn)
IGP46	GP7 / GPn* 100	The percentage of G1 glycan in total neutral IgG glycans (GPn)
IGP47	GP8 / GPn* 100	The percentage of G1[6]F glycan in total neutral IgG glycans (GPn)
IGP48	GP9 / GPn* 100	The percentage of G1[3]F glycan in total neutral IgG glycans (GPn)
IGP49	GP10 / GPn* 100	The percentage of G1[6]FN glycan in total neutral IgG glycans (GPn)
IGP50	GP11 / GPn* 100	The percentage of G1[3]FN glycan in total neutral IgG glycans (GPn)
IGP51	GP12 / GPn* 100	The percentage of G2 glycan in total neutral IgG glycans (GPn)
IGP52	GP13 / GPn* 100	The percentage of G2N glycan in total neutral IgG glycans (GPn)

IGP53	GP14 / GPn* 100	The percentage of G2F glycan in total neutral IgG glycans (GPn)
IGP54	GP15 / GPn* 100	The percentage of G2FN glycan in total neutral IgG glycans (GPn)
IGP55	SUM(GP1n: GP4n + GP6n)	The percentage of agalactosylated structures in total neutral IgG glycans
IGP56	SUM(GP7n: GP11n)	The percentage of monogalactosylated structures in total neutral IgG glycans
IGP57	SUM(GP12n: GP15n)	The percentage of digalactosylated structures in total neutral IgG glycans
IGP58	SUM(GP1n+ GP4n+ GP6n+ GP8n+ GP9n+ GP10n+ GP11n+ GP14n+ GP15n)	The percentage of all fucosylated structures (+/- bisecting GlcNAc) in total neutral IgG glycans
IGP59	SUM(GP1n+ GP4n+ GP6n) / G0n * 100	The percentage of fucosylation of agalactosylated structures in total neutral IgG glycans
IGP60	SUM(GP8n+ GP9n+ GP10n+ GP11n) / G1n * 100	The percentage of fucosylation of monogalactosylated structures in total neutral IgG glycans
IGP61	SUM(GP14n+ GP15) / G2n * 100	The percentage of fucosylation of digalactosylated structures in total neutral IgG glycans
IGP62	SUM(GP1n+ GP4n+ GP8n+ GP9n+ GP14n)	The percentage of fucosylated structures (without bisecting GlcNAc) in total neutral IgG glycans
IGP63	SUM(GP1n+ GP4n) / G0n * 100	The percentage of fucosylation of agalactosylated structures (without bisecting GlcNAc) in total neutral IgG glycans
IGP64	SUM(GP8n+ GP9n) / G1n * 100	The percentage of fucosylation of monogalactosylated structures (without bisecting GlcNAc) in total neutral IgG glycans
IGP65	GP14n/ G2n * 100	The percentage of fucosylation of digalactosylated structures (without bisecting GlcNAc) in total neutral IgG glycans
IGP66	SUM(GP6n + GP10n + GP11n + GP15n)	The percentage of fucosylated structures (with bisecting GlcNAc) in total neutral IgG glycans
IGP67	GP6n/ G0n * 100	The percentage of fucosylation of agalactosylated structures (with bisecting GlcNAc) in total neutral IgG glycans
IGP68	SUM(GP10n + GP11n) / G1n * 100	The percentage of fucosylation of monogalactosylated structures (with bisecting GlcNAc) in total neutral IgG glycans
IGP69	GP15n / G2n * 100	The percentage of fucosylation of digalactosylated structures (with bisecting GlcNAc) in total neutral IgG glycans
IGP70	FBn/ Fn * 100	Ratio of fucosylated structures with and without bisecting GlcNAc in total neutral IgG glycans
IGP71	FBn/ Fn total * 100	The incidence of bisecting GlcNAc in all fucosylated structures in total neutral IgG glycans
IGP72	Fn/(GP13n + FBn)	Ratio of fucosylated non-bisecting GlcNAc structures and all structures with bisecting GlcNAc in total neutral IgG glycans
IGP73	GP13n/ (Fn+ FBn) * 1000	Ratio of structures with bisecting GlcNAc and all fucosylated structures (+/- bisecting GlcNAc) in total neutral IgG glycans
IGP74	GP15n/GP14n	Ratio of fucosylated digalactosylated structures with and

		without bisecting GlcNAc in total neutral IgG glycans
IGP75	GP15n/(GP14n + GP15n) * 100	The incidence of bisecting GlcNAc in all fucosylated digalactosylated structures in total neutral IgG glycans
IGP76	GP14n/(GP13n + GP15n)	Ratio of fucosylated digalactosylated non-bisecting GlcNAc structures and all digalactosylated structures with bisecting GlcNAc in total neutral IgG glycans
IGP77	GP13n/(GP14n + GP15n) * 1000	Ratio of digalactosylated structures with bisecting GlcNAc and all fucosylated digalactosylated structures (+/- bisecting GlcNAc) in total neutral IgG glycans

Genome-wide association studies

Prior to analyses, each cohort received quality controlled glycan data (glycan data were pre-processed by the phenotype provider) and an analysis plan for genome-wide association studies. GWAS were performed on age, sex and cohort specific covariate corrected glycan data and imputed genotypes, taking into account relatedness and population stratification and assuming an additive model of association.

Two independent analysts processed each of 77 individual-level GWAS per cohort based on a protocol suggested by Winkler *et al* (*38*) and involved removing uninformative and SNPs of low imputation quality (rsq_hat < 0.3 and MACH (*63*)), proper info <0.4 (IMPUTE (*64*, *65*)), as well as verification of trait transformations. A summary of quality control outcomes can be seen in Appendix Table 6.

Each cleaned set of individual GWA studies was pooled independently by two analysts. Meta-analyses were performed using fixed effect inverse-variance method implemented in METAL (*39*). Effect estimates and p-values of the final meta-analyses were compared and showed no inconsistencies.

Prior to meta-analysis, each GWA study was corrected for genomic control inflation factor. The genomic control inflation factor λ varied from 0.95 to 1.05, suggesting little residual influence of population stratification. Additional genomic control was performed on the aggregated meta-analysis results.

Appendix Table 5. Details of genome-wide association analyses.

Cohort	Method used for correction for genetic substructure	Covariates	GWAS Software
TwinsUK	Linear mixed models (LMM) with kinship matrix	Age, sex	SNPTEST
ORCADES	LMM with kinship matrix	age, sex, genotyping chip	ProbABEL
CROATIA-Korcula	LMM with kinship matrix	age, sex	ProbABEL
CROATIA-Vis	LMM with kinship matrix	age, sex	ProbABEL
EGCUT	5 first principle components		SNPTEST
FINRISK	First 10 genomic PCA dimensions tested for association with endpoint, all with P > 0.05, thus omitted from the final analysis	age+sex+genotyping batch+IBD+RA+CC	SNPTEST
COGS	None needed	Age, sex	SNPTEST
SDRNT1BIO	None needed	Age, sex	SNPTEST
Leiden Longevity Study	LMM with kinship matrix	Age, sex	QT-assoc

Appendix Table 6. Individual GWAS file-level quality control.

Showing the results for only one random glycan given that all 77 glycan files in every cohort had the same number of SNPs removed. Shown are only filters that removed at least 1 SNP.

cohort	numSNPsIn	numSNPsOut	Monomorph	Missing P	MAClet6	Low Information
TwinsUK	2130725	1939891	0	0	167937	22897
ORCADES	2505100	2477038	671	0	22509	4882
CROATIA-Korcula	2543887	2392625	142	11623	18955	120542
CROATIA-Vis	2543887	2448535	192	1465	29725	63970

numSNPsIn – number of SNPs before QC; numSNPsOut - number of SNPs after QC; Monomorph – number of SNPs excluded because they were monomorphic (EAF =1 or EAF = 0); missing p – number of SNPs excluded because of the missing p-value; MAClet6 – number of SNPs removed because minor allele count was smaller than 6; Low information – number of SNPs excluded because of the poor quality

Validation Genome-wide association studies

Additional validation of findings was performed by running GWAS on 1841 samples from the Leiden Longevity Study, measured with liquid chromatography electrospray mass spectrometry (LCMS) as previously described (*66*). Immunoglobulin G has two regions – Fab region that interacts with antigens and Fc region that interacts with effector cells of the immune system. Both regions can have covalently bound glycans. IgG molecules can further

be divided in 4 subclasses, each with different structure and function (*67*). While UPLC provides information of total IgG glycosylation (glycans bound to both Fab and Fc regions of IgG), LCMS quantifies only glycans bound to the Fc region. As a result, to fully replicate an UPLC finding, one would need to combine results from three different glycan traits measured with LCMS (IgG2 and IgG3 are not distinguishable with LCMS). For example, to replicate association with G0 glycan in total IgG glycans (UPLC trait IGP2), one would need to combine association results from three LCMS glycan traits – namely IgG1_G0, IgG2_G0 and IgG4_G0), with a caveat that UPLC G0 glycan trait contains information on both Fab and Fc G0 glycan, while the three LCMS traits will contain information of only Fc G0 glycan levels. In addition, not all UPLC measured glycans have their counterpart in LCMS data. For example, association of UPLC IGP35 (ratio of mono- and di-sialylated glycans) and rs7700895 cannot be validated with LCMS data since disialylated glycans are bound only to the Fab region, whose glycosylation is not measured with the LCMS method (*68*). Given these differences between discovery and validation datasets, we aimed at replicating association of the top SNP in every locus with any LCMS glycan trait, aiming at validating association with the locus rather than specific glycan-SNP pair. Genome-wide association studies were ran for both 50 directly measured and 155 derived LCMS glycan traits and SNPs mapping to 27 significantly associated loci. Given that 155 derived traits are linear combination of the 50 directly measured traits we set the validation significance level top ≤ 3.7e-05 (Bonferroni correction for 50 glycan traits and 27 loci). More details about the LMCS traits that were significantly associated with the top SNPs can be seen in Appendix Table 7.

Appendix Table 7. Description of structures for LCMS glycans. Adapted from Huffman *et al*(*46*)

GWAS Name	Trait/Formula	DESCRIPTION
**Measured glycan traits**		
LC_IGP7	IgG1_G1FS1	The percentage of FA2G1S1 glycan in total IgG1 glycans
LC_IGP11	IgG1_G0	The percentage of A2glycan in total IgG1 glycans
LC_IGP19	IgG1_G1NS1	The percentage of A2BG1S1 glycan in total IgG1 glycans
LC_IGP92	IgG2_G2FN	The percentage of FA2BG2 glycan in total IgG2 glycans
LC_IGP180	IgG4_G2FS1	The percentage of FA2G2S1 glycan in total IgG4 glycans
**Derived traits**		
LC_IGP27	IgG1 SUM(G1S1+G2S1)/ SUM(G0+G1+G1S1+G2+G2S1)*100	The percentage of monosialylation of all afucosylated structures without bisecting GlcNAc in total IgG1 glycans
LC_IGP34	IgG1 SUM(G1FS1+G2FS1)/ SUM(G1F+G1FS1+G2F+G2FS1)*100	The percentage of monosialylation of fucosylated galactosylated structures without bisecting GlcNAc in total IgG1 glycans
LC_IGP40	IgG1 FBG1S1/(FBG1+FBG1S1)	The percentage of monosialylation of fucosylated monogalactosylated (with bisecting GlcNAc) structures in total IgG1 glycans
LC_IGP48	IgG1_G2Fn/IgG1 GPn*100	The percentage of FA2G2 glycan in neutral IgG1 glycans
LC_IGP63	IgG1 SUM(G1Fn+G1FNn)/G1n*100	The percentage of fucosylation of monogalactosylated structures in neutral IgG1 glycan fraction
LC_IGP76	IgG1 SUM(G2Nn+G2FNn)/G2n*100	The incidence of bisecting GlcNAc (+/- core Fuc) in digalactosylated structures in neutral IgG1 glycan fraction
LC_IGP81	IgG1 Fn/Bn	Ratio of fucosylated structures without bisecting GlcNAc and afucosylated structures with bisecting GlcNAc in neutral IgG1 glycan fraction
LC_IGP84	IgG1 FBn/Bn total*100	Ratio of fucosylated structures without bisecting GlcNAc and afucosylated structures with bisecting GlcNAc in neutral IgG1 glycan fraction
LC_IGP113	IgG2 SUM(G1S1+G2S1)/ SUM(G0+G1+G1S1+G2+G2S1)*100	The percentage of monosialylation of all afucosylated structures without bisecting GlcNAc in total IgG2 glycans
LC_IGP115	IgG2 G2S1/SUM(G2+G2S1)*100	The percentage of monosialylation of afucosylated digalactosylated (without bisecting GlcNAc) structures in total IgG2 glycans
LC_IGP122	IgG2 G1FS1/SUM(G1F+G1FS1)*100	The percentage of monosialylation of fucosylated monogalactosylated (without bisecting GlcNAc) structures in total IgG2 glycans
LC_IGP133	IgG2 IgG2_G1Fn/IgG2 GPn*100	The percentage of FA2G1 glycan in neutral IgG2 glycans
LC_IGP154	IgG2 G2Fn/G2n*100	The percentage of fucosylation (without bisecting GlcNAc) of digalactosylated structures in neutral IgG2 glycan fraction
LC_IGP155	IgG2 SUM(G0FNn+G1FNn+G2FNn)	The percentage of fucosylated (with bisecting GlcNAc) structures in neutral IgG2 glycan fraction
LC_IGP156	IgG2 G0FNn/G0n*100	The percentage of fucosylation (with bisecting GlcNAc) of agalactosylated structures in neutral IgG2 glycan fraction
LC_IGP161	IgG2 SUM(G1Nn+G1FNn)/G1n*100	The incidence of bisecting GlcNAc (+/- core Fuc) in monogalactosylated structures in neutral IgG2 glycan fraction
LC_IGP183	IgG4 SUM(G0FN+G1FN+G2FN)	The incidence of bisecting GlcNAc of IgG4

LC_IGP187	IgG4 SUM(G1FS1+G2FS1)/ SUM(G1F+G1FS1+G2F+G2FS1)*100	The percentage of monosialylation of fucosylated galactosylated structures without bisecting GlcNAc in total IgG4 glycans
LC_IGP188	IgG4 SUM(G1FS1+G2FS1)/ SUM(G0F+G1F+G1FS1+G2F+G2FS1)*100	The percentage of monosialylation of all fucosylated structures without bisecting GlcNAc in total IgG4 glycans
LC_IGP201	IgG4 IgG4_G1FNn/IgG4 GPn*100	The percentage of FA2BG1 glycan in neutral IgG4 glycans

SMR/HEIDI analysis for pleiotropy with gene expression and complex traits

We used Summary data-based Mendelian Randomization (SMR) analysis followed by the Heterogeneity in Dependent Instruments (HEIDI) test to identify potential pleiotropic effects of identified loci on glycan levels and other complex traits including gene expression levels in certain tissues (*12*). SMR analysis provides evidence for pleiotropy, but is unable to define whether both traits are affected by the same underlying causal polymorphism. The latter is specified by a HEIDI test that distinguishes pleiotropy from linkage disequilibrium (indicating that there are two distinct causal variants, in linkage disequilibrium, but each regulating different trait). We started by selecting the top genome-wide significant SNP for each glycan in every locus and checked if association results for specific probe/complex trait are reported in the region of interest. List of the tested complex traits can be seen in the Appendix Table 8.

In our implementation of the HEIDI test we first compute statistic $T_H = \sum_i^m z_{d(i)}^2$, where m is the number of markers selected for the test and $z_{d(i)}$ is the scaled measure of deviation of the ratio of the effect estimates from the two GWAS from that at the top associated SNP (see Zhu et al. work for details (*12*)). LD matrix (r2) was computed with PLINK 1.9 using 1000 Genomes data for 503 European individuals (http://www.internationalgenome.org/data/). Up to 20 SNPs have been selected for this test using the following procedure:

1) Define eligible SNPs in the region +/- 250 kb from the top primary GWAS association, by selecting those that have $\chi^2 > 10$ in primary GWAS, and have results reported in secondary GWAS

2) Make empty "target" (S_t) and "rejected" (S_r) SNP sets

3) Select current SNP with the lowest p from the primary GWAS that is neither in the target nor in the rejected set

4) If current SNPs has r^2>0.9 (computed with PLINK 1.9) with any SNP in the target SNP set or MAF≤0.03, add current SNP to the rejected set

5) Otherwise add current SNP to the target set

6) Repeat from the third step until either the eligible SNP set is exhausted or the target set has 20 SNPs

7) Use target set for HEIDI test

In case the number of SNPs was less than 3, no test was performed. The method was implemented using Python 3.5 as the main programming language, Pandas 1.19.2 (https://conference.scipy.org/proceedings/scipy2010/mckinney.html) for data processing, Dask 0.14.1 (http://dask.pydata.org/) for distributed data pre-processing, Bokeh 1.12.4 (http://bokeh.pydata.org/) for graphics.

Appendix Table 8. Summary-level statistics downloaded for SMR or HEIDI test.

Trait	short name	Link to data	reference
Ankylosing spondylitis	spondylitis	https://www.immunobase.org/downloads/protected_data/iChip_Data/hg19_gwas_ic_as_igas_4_19_1.tab.gz	Cortes et al (*69*)
Asthma	asthma	https://grasp.nhlbi.nih.gov/downloads/ResultsOctober2016/Moffatt/asthma_gabriel_results.zip	Moffatt et al (*70*)
Crohn's Disease	crohn	https://www.ibdgenetics.org/downloads.html	Liu et al (*71*)
HDL	hdl	http://csg.sph.umich.edu/abecasis/public/lipids2013/	Willer et al (*72*)
Height	height	http://portals.broadinstitute.org/collaboration/giant/index.php/GIANT_consortium_data_files	Wood et al (*73*)
Inflammatory bowel disease	ibd	https://www.ibdgenetics.org/downloads.html	Liu et al (*71*)
Parkinson's Disease	parkinson	https://grasp.nhlbi.nih.gov/downloads/ResultsOctober2016/Pankratz/Pankratz_Parkinsons_22687-SuppTable1.txt	Pankratz et al (*74*)
Primary biliary cirrhosis	cirrhosis	https://www.immunobase.org/downloads/protected_data/GWAS_Data/hg19_gwas_pbc_cordell_4_20_0.tab	Cordell et al (*75*)
Ulcerative colitis	uc	ftp://ftp.sanger.ac.uk/pub/consortia/ibdgenetics/ucmeta-sumstats.txt.gz	Liu et al (*71*)
Rheumatoid Arthritis	ra	https://grasp.nhlbi.nih.gov/downloads/ResultsOctober2016/Okada/RA_GWASmeta_European_v2.txt.gz	Okada et al (*76*)

Functional network of genes associated with IgG N-glycosylation

Only 23 glycans are directly measured and all others are a function of these measured traits and are, therefore, correlated to different extent. Especially high phenotypic correlation can be observed between IGP1-IG14 and IGP41-IGP54 (Appendix Figure 1). These traits represent the same glycan traits but normalised in a different fashion. Since charged glycans are more prone to experimental error, glycan traits IGP41-IGP54 are defined as IGP1-IGP15 normalised by the total area of these peaks (instead of the total area of the whole chromatogram). Because of the redundancy of these glycan traits, they were removed prior the association analysis. Permutation analysis was then performed to assess the impact of correlations between remaining glycans on the glycome-wide pairwise SNP effect

associations and additionally validate the observed network edges as described in the main Methods.

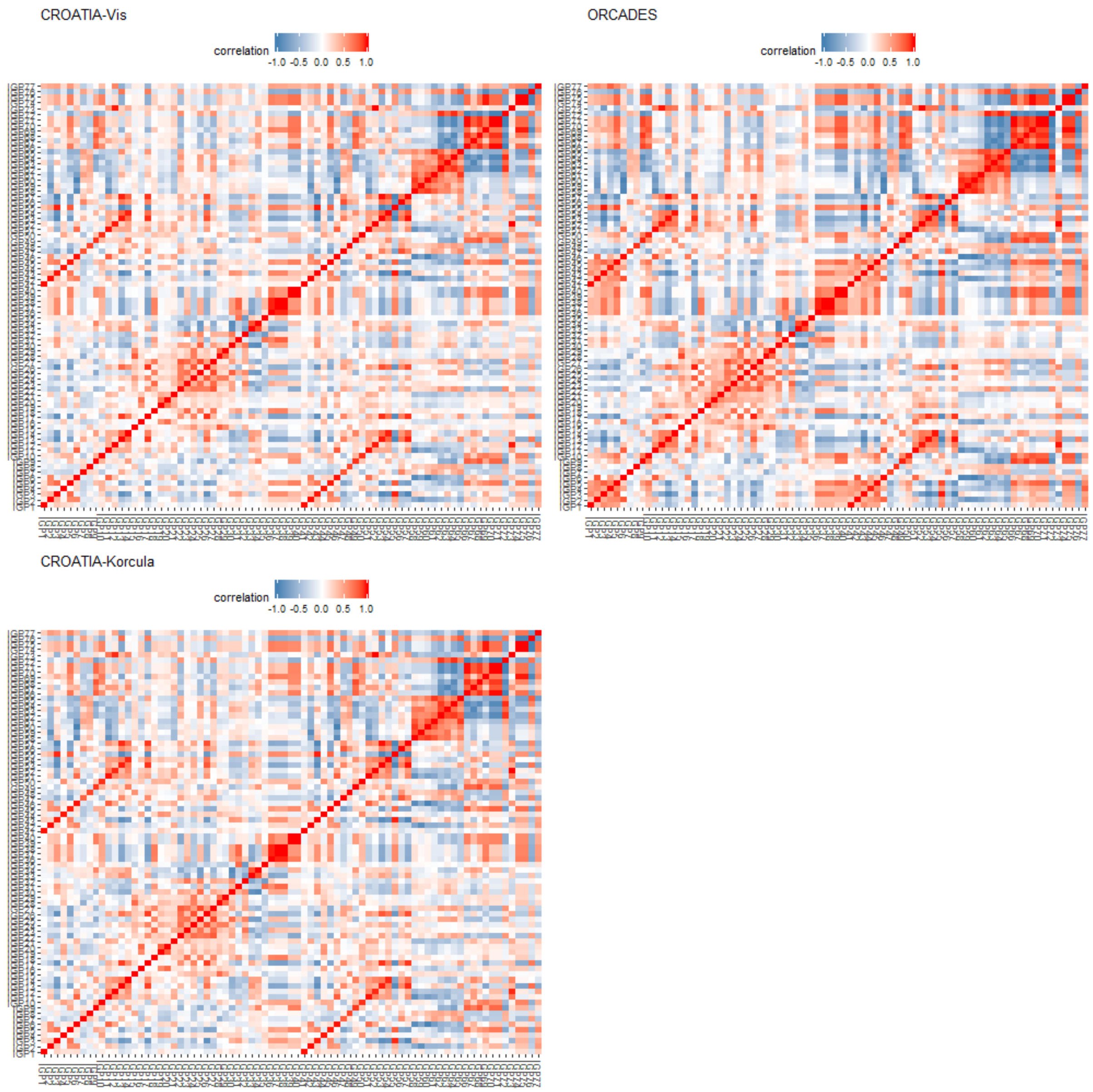

Appendix Figure 1. Phenotypic correlation of UPLC IgG *N*-glycans.

ChIP-seq data (.narrowPeak files) for the transcription factors (TFs) that we reported as associated with IgG glycosylation (Supplementary Table 4) was downloaded from http://hgdownload.cse.ucsc.edu/goldenPath/hg19/encodeDCC/ for the GM12878 lymphoblastoid cell line. For transcription factors that are not available in the ENCODE data the PWMs used were obtained from previously known motifs in the ENCODE (*77*), JASPAR (*78*) and Hocomoco (*79*) databases. The peak-motifs tool (*18*) in Regulatory Sequence

Analysis Tools (RSAT) was used for motif discovery in the peak sequences of ChIP-seq datasets.

Allele specific binding of the TFs was tested using the matrix-scan tool (*80*) in RSAT. Sequences surrounding the top SNPs from the GWAS were obtained from ensemble (*81*). Position weight matrices (PWMs) of the discovered motifs outputted from peak-motifs were used to determine whether these SNPs fall within the binding sites of the TFs. The same analysis was performed for all significantly and suggestively associated IgG glycosylation SNPs (N=1723) and non-associated SNPs within 50kb of glycosylation loci (N=4389).

Allele specific binding was estimated by comparing binding weights of the motifs to the sequence were between reference and alternate alleles. A SNP was considered to influence TF binding if one of the alleles significantly affected its binding (p$\leq 0.05/2764712 \sim 1.8 \times 10^{-8}$, corrected for the total number of performed tests).

In vitro* validation of the functional links between *IKZF1* and *FUT8

ShRNA cloning, cell culture, transfections and shRNA knockdown

Four short hairpin RNAs (shRNA) targeting the coding region or 3’ UTR of all *IKZF1* isoforms were designed using BlockIT RNAi Designer (https://rnaidesigner.thermofisher.com/rnaiexpress/). Single stranded oligos for IKZF1 shRNA and a sequence with no corresponding target in the human genome (random shRNA, 5’-GATCATGTAGATACGCTCA-3’) were annealed and cloned into pENTR/H1/TO vector using the BLOCK-iT™ Inducible H1 RNAi Entry vector kit (Invitrogen). The vectors were propagated, sequenced and used for electroporation of an IgG1-secreting human lymphoblastoid cell line (LCL), MATAT6. MATAT6 cells were maintained in RPMI1640

medium supplemented with 10% fetal calf serum (FCS), 4 mM L-glutamine, 1 mM oxaloacetate, 0.45 mM sodium pyruvate, 1.25 mM MOPS, bovine insulin, 100 U/ml penicillin/streptomycin. All experiments were performed on exponentially growing cells maintained at concentrations <1 × 10^6/ml. Cells were electroporated with pENTR-shRNA plasmids using Neon Transfection system (Invitrogen). Electroporation parameters were 1300 V/ 20 ms/ 2 pulses. For each construct, stable bulk cultures constitutively expressing the shRNA were selected with 50 µg/ml zeocin for 14 days prior to removal of antibiotic. RNA was extracted to assess gene knockdown efficiency for each shRNA tested by qPCR, and the IKZF1 shRNA (5'-GGTAAAGTCCTAGCACCAATT-3') resulting in most significant knockdown was used for subsequent experiments. Stable bulk cultures were generated from a minimum of 2 independent electroporation reactions.

RNA, cDNA and Real-time PCR

RNA was extracted from MATAT6 cells and stable shRNA lines using the RNeasy Mini kit (Qiagen), following the manufacturer's instructions and supplied buffers. On-column DNase digestion was performed. cDNA was synthesised using Transcriptor High Fidelity cDNA Synthesis Kit (Roche). qPCR was performed using TaqMan Universal PCR Mastermix, No AmpErase UNG, and gene expression assays for *IKZF1* (Hs00958473_m1), *FUT8* (Hs00189535_m1), *IKZF3* (Hs00232635_m1) and *HPRT1* (Hs02800695_m1) on the Applied Biosystems HT7900. Samples were run in triplicate. Relative gene expression level was determined using the comparative Ct method. Error bars show standard deviation obtained from a minimum of 3 experiments. Statistically significant differences in gene expression were determined using paired t-test.

Western blot analysis

Whole cell lysates were prepared by lysing cells in RIPA Buffer (25 mM Tris-HCl, pH 7.5, 150 mM NaCl, 1% NP-40, 1% sodium deoxycholate, 0.1% SDS) on ice for 1 hour. Jurkat nuclear extract (SC2131, Santa Cruz Biotechnologies) and HEK293T lysates were used as positive and negative controls for IKZF1. Protein concentration was determined by Bradford Assay (Pierce). 10 μg of total protein was reduced and denatured prior to separation on 4-12% Bis-Tris NuPAGE gel (Invitrogen) in MOPS Buffer (Invitrogen), before transfer to PVDF membrane. Membranes were blocked in 5% non-fat dried milk in PBST (phosphate buffered saline, 0.5% Tween 20), and probed using the following antibodies: rabbit polyclonal anti-Ikaros (H-100, Santa Cruz Biotechnology), mouse anti-alpha tubulin (Sigma Alrich). Secondary antibodies were horseradish peroxidase conjugated anti-rabbit IgG, or anti-mouse IgG (Sigma Aldrich). Antibody detection was achieved using the enhanced chemiluminescent (ECL) detection system (Amersham Biosciences, UK).

Chromatin Immunoprecipitation (ChIP)

ChIP assays were performed using ExactaChIP buffers (R&D Systems), as described by the manufacturer except for the following modifications. MATAT6 cells cultured at 1×10^6 cells/ml of media were collected by centrifugation and resuspended in fresh media. Cells were cross-linked in a final formaldehyde concentration of 1% (w/v) for 5 minutes, followed by the addition of glycine to 125mM and incubation for 10 min. After washing with cold PBS, 5×10^6 cells were sonicated 15 times in 30 s pulses at maximum power in 500 μl Lysis Buffer (ExactaChIP, R&D Systems) and Complete EDTA-free protease inhibitor (Roche) to shear chromatin to an average length of 500 bp. Lysates were cleared by centrifugation for 10 minutes at 12,000 *g*, and then the supernatant was diluted in Dilution Buffer (ExactaChIP, R&D Systems). ChIP input was incubated with either anti-IKZF1 (AF4984, R&D Systems) or Goat IgG Isotype Control Antibody (AB108C, R&D Systems) overnight at 4ºC with

rotation. 5 μg of biotinylated anti-goat IgG (BAF109, R&D Systems) was added for a further 2 hours, prior to the addition of Streptavidin-agarose beads (Sigma Aldrich) for 1 hour. Agarose beads were collected by centrifugation, and sequential washes performed with Wash Buffer 1 – Wash Buffer 4 (R&D Systems). After the final wash, Chelating Resin solution was added to the beads and the samples boiled for 10 minutes. Immunoprecipitated DNA or input material was incubated at 65°C to remove cross-links, and purified using the Qiagen PCR purification kit. ChIP-PCR was performed using primers flanking a binding site upstream of *FUT8* identified by ChIP-Seq analysis in the GM12878 cell line (Forward, 5' - ACAGGGAAGATGAGTGGGAG- 3', Reverse 5' – AGGACTTCTGGGACTTCGTC- 3') to confirm Ikaros-DNA complexes also occur in MATAT6 cells. PCR products were analysed on 2% (w/v) agarose gel by electrophoresis in Tris-borate EDTA buffer.

Results

Prioritising genes associated with IgG N-glycosylation

First, we investigated each candidate gene's functional similarity to members of enriched gene-sets. Data-driven Expression-Prioritized Integration for Complex Traits (DEPICT) (*10*) significantly prioritised genes in 14 loci at FDR ≤ 0.05 and another 3 at FDR ≤ 0.2 (Appendix Table 9). Five loci span glycosyltransferase genes and three of these were selected using DEPICT (*ST6GAL1* and *B4GALT1* at FDR ≤ 0.05 and *FUT8* at FDR ≤ 0.2). In the remaining loci prioritised genes (*IRF1*, *NXPE4*, *IKZF1*, *IKZF3*, *ORMDL3*, *GSDMB*, *GGA2*, *ARHGAP27*, *RUNX1*, *VPREB3* at FDR ≤ 0.05 and *RUNX3*, *SLC38A10* at FDR ≤ 0.2) have no known role in protein glycosylation but are predicted to be involved in pathways and tissues related to development and activation of B-cells, the cells responsible for synthesis of IgG. Full DEPICT results can be seen in Appendix Table 9.

We next checked if any of the associated SNPs (both significant and suggestive – 1,815 SNPs in total) result in a change in the amino acid (AA) sequence and, therefore, potentially have a more direct influence on the function of the resulting protein. Using the PolyPhen-2 and SIFT (*82*) tools (as implemented in VEP (*11*)) we found that genes from 7 loci contain non-synonymous mutations, with at least one probably damaging variant in genes from 2 loci and non-sense variants in one locus. SNPs coding for tolerated AA changes were observed in *NXPE1* (rs7944960:Leu>Ser), *NXPE4* (rs550897:Tyr>His), *SLC22A4* (rs1050152:Leu>Phe), *ZPBP2* (rs11557467:Ser>Ile) and *SYNGR1* (rs4821888:Thr>Ala). Probably damaging AA changes were observed in *GSDMB* (rs2305479:Gly>Arg), *SPPL2C* (rs12185233:His>Pro, rs12373142:Pro>Arg) and *MAPT* (rs17651549:Arg>Trp). The last three SNPs are in high LD, making it impossible to distinguish the most likely target gene in this locus. In addition, IgG *N*-glycosylation associated variants in *CRHR1* and *MAPT* IgG *N*-glycosylation

associated variants result in non-sense mutations (*CRHR1* rs16940665:stop>Arg, *MAPT* rs754512:Lys>stop) (Appendix Table 10).

The final step was to check if any of the glycosylation associated SNPs were previously associated with gene expression. Given that IgGs are predominantly synthesised in B-cells, often activated by a signal from other immune cells, we looked at expression QTLs (eQTLs) from 5 different immune cells (B-cells, two types of T-cells, neutrophils and macrophages) from the CEDAR dataset (N=350; Momozawa *et al* (*13*)) and peripheral blood from Westra *et al* (*14*) (N=5,311). To extend the analysis beyond a single shared SNP, we applied Summary-level Mendelian Randomization (SMR) with Heterogeneity in Dependent Instruments (HEIDI) (*12*).

In total, in 14 genome-wide significant loci we observed significant SMR ($p_{SMR} \leq 0.05/2{,}622 = 1.9\times10^{-5}$) for expression with at least one glycan trait. In 9 of these loci we observed pleiotropy of IgG glycosylation with gene expression ($p_{HEIDI} \geq 0.05$). In CD19 B-cells, the cell lineage primarily responsible for synthesis of IgG, pleiotropy was observed with expression of four genes (*B4GALT1*, *GSDMB*, *ORMDL3*, *MGAT3*) (Figure 1, https://shiny.igmm.ed.ac.uk/igg_glycans_gwas/). Two of these genes, *MGAT3* and *B4GALT1*, are glycosyltransferases. The remaining two genes, *ORMDL3* and *GSDMB*, have no known direct link with glycosylation, but were in the past strongly linked to asthma, inflammatory bowel disease (IBD) and other immune diseases (*83*). In peripheral blood we observed pleiotropy with expression of 8 additional genes (glycosyltransferase *ST6GAL1* and *SLC22A4*, *COG7*, *CEP131*, *TEPSIN*, *SLC38A10*, *RFXANK*, *CHCHD10*). The lack of pleiotropy with expression of glycosyltransferases *FUT8* and *ST6GAL1* in B-cells can partly be explained by weak eQTLs for these genes (the lowest observed eQTL p-values for *ST6GAL1* in B-cells = 3.5×10^{-4} and 3.62×10^{-4} for *FUT8*, Appendix Table 11). Full

results of this analysis can be seen in the online resource available at https://shiny.igmm.ed.ac.uk/igg_glycans_gwas/.

Additional validation of the functional network regulating IgG *N*-glycosylation

We hypothesised that the transcription factors in the functional network may be involved directly in regulation of the expression of glycosyltransferase genes in IgG secreting cells. The regulatory potential of the associated variants in B-cells, and the impact upon transcription factor binding, was inferred by interrogation of ENCODE chromatin-immunoprecitation sequencing (ChIP-seq) experiments performed in the lymphoblastoid cell line (LCL) GM12878, or by comparison to known binding site motifs. As shown in Supplementary Table 9, a striking number of the variants tested were predicted to have strong impact on binding weight scores obtained for RUNX1, RUNX3 and IRF1, providing further evidence to corroborate the links in the functional network in Figure 2a.

FUT8 had the strongest glycome-wide association with the *ORMDL3-GSDMB-IKZF3-ZPBP2* and *IKZF1* loci. Unlike rs5750830, the lead SNP for the *MGAT3* loci, we found no evidence that the six variants contributing to variation in IGP42 glycan level in the region of *FUT8* (Supplementary Table 3) are eQTLs for *FUT8*, or that they alter binding sites for IKZF1 using RSAT for motif discovery in GM12878 Encode IKZF1 Chip-Seq data. We next identified IKZF1 binding sites on chromosome 14 near to FUT8 in the GM12878 Encode ChIP-Seq data, and performed chromatin immunoprecipitation (ChIP) assays to investigate occupancy of these sites. In the GM12878 Encode ChIP-Seq data, 10 sites upstream of FUT8 were occupied by IKZF1. In an IgG secreting LCL, IKZF1 bound most strongly to chr14: 65757755-65758024 (Figure 3a).

Interrogation of sequences surrounding SNPs in high LD with rs11847263 (top *FUT8* SNP): rs1054218, rs8006608, rs4073416, rs4902409 or rs4400971 predicted that variants on the same haplotype will affect IRF, RUNX1, RUNX3 and CTCF binding sites (Supplementary Table 9). Due to the low frequency of this haplotype, we were unable to identify a lymphoblastoid cell line (LCL) with appropriate genotype to assess the functional consequences of these putative alterations in transcription factor and CTCF binding. Publically available Hi-C profiling (*45*) performed to 1 kb resolution in the LCL GM12878 indicates that these SNPs lie in the same chromosomal topological associating domain (TAD) as the transcription start site (TSS) of *FUT8* (Supplementary Figure 3). CTCF plays a crucial role in maintaining the 3-dimensional structure of the genome, and disruption of CTCF binding by SNPs in LD with rs4400971 may modify the interactions formed between enhancer and promoter regions of *FUT8* to affect expression of the glycosyltransferase enzyme.

Appendix Table 9. DEPICT gene prioritization.

SNP - SNP with the lowest p-value with any of the glycans from GCTA-joint analysis; SNP P-value - p-value of the SNP in GCTA-joint analysis; gene - HUGO gene symbol; Nominal p-value - the nominal gene prioritisation p-value; FDR - the estimated false discovery rate of the gene

Locus	SNP	# genes in locus	GWAS P-value	Gene	Enrichment P-value	Closest to lead SNP	FDR
1:25226001-25345011	rs10903118	1	2.41e-12	RUNX3	3.00e-02	true	<0.20
1:61362132-61374136	rs17303508	1	3.82e-08	NFIA	3.50e-01	true	>0.20
1:246854862-246963137	rs3795464	2	1.06e-08	SCCPDH	8.40e-01	false	>0.20
				ENSG00000227953	9.00e-01	true	>0.20
2:100636757-	rs2309748	2	3.46e-09	AFF3	1.90e-01	false	>0.20

100805273				ENSG00000230393	6.40e-01	true	>0.20
3:186712711-186738421	rs7621161	1	2.82e-162	ST6GAL1	1.24e-03	true	<0.05
5:95211647-95347786	rs7700895	1	1.48e-14	ELL2	9.00e-02	true	>0.20
5:131026218-131833599	rs11748193	11	4.53e-10	IRF1	7.89e-03	false	<0.05
				IL3	8.78e-03	false	<0.05
				CSF2	5.00e-02	false	<0.20
				FNIP1	9.00e-02	false	>0.20
				C5orf56	3.40e-01	false	>0.20
				SLC22A4	4.60e-01	false	>0.20
				P4HA2	6.20e-01	false	>0.20
				PDLIM4	8.80e-01	false	>0.20
				SLC22A5	9.50e-01	false	>0.20
				ACSL6	9.80e-01	false	>0.20
				ENSG00000233006	1.00e+00	true	>0.20
6:139617590-139636003	rs9385856	1	5.99e-19	TXLNB	5.00e-01	true	>0.20
6:143150223-143203591	rs7758383	1	1.11e-13	HIVEP2	2.20e-01	true	>0.20
7:6520676-6537913	rs6964421	2	6.17e-11	KDELR2	2.60e-01	false	>0.20
				DAGLB	9.30e-01	true	>0.20
7:50325717-50361683	rs6421315	1	1.22e-26	IKZF1	2.10e-03	true	<0.05
7:150906453-150969535	rs7812088	2	3.52e-22	ABCF2	3.30e-01	true	>0.20
				SMARCD3	9.60e-01	false	>0.20
8:103538266-103550211	rs10096810	1	1.12e-10	ODF1	9.10e-01	true	>0.20
9:33041761-33186080	rs10813951	1	1.77e-49	B4GALT1	1.00e-02	true	<0.05
9:33205136-33375592	rs12341905	4	3.56e-08	BAG1	3.90e-01	false	>0.20
				NFX1	5.20e-01	false	>0.20
				SPINK4	8.30e-01	true	>0.20
				CHMP5	8.70e-01	false	>0.20
11:114323627-114450529	rs481080	2	1.23e-16	NXPE4	3.30e-03	false	<0.05
				NXPE1	3.90e-01	true	>0.20
14:65472891-66284991	rs10148907	1	1.05e-55	FUT8	2.00e-02	true	<0.20
14:105966019-106002352	rs4074453	1	7.66e-29	TMEM121	4.60e-01	true	>0.20
16:23397113-23613191	rs250555	5	7.83e-10	GGA2	9.09e-04	false	<0.05
				NDUFAB1	6.00e-02	false	<0.20
				UBFD1	1.00e-01	false	>0.20
				EARS2	1.90e-01	false	>0.20
				COG7	5.30e-01	true	>0.20
17:16820099-16875636	rs4561508	2	6.14e-09	TNFRSF13B	1.88e-04	false	<0.05
				TBC1D27	6.70e-03	true	<0.05

17:37903731-38112190	rs7216389	4	1.74e-15	IKZF3	2.23e-03	false	<0.05
				ORMDL3	3.76e-04	false	<0.05
				GSDMB	8.90e-03	true	<0.05
				ZPBP2	7.30e-01	false	>0.20
17:43463492-44896083	rs199456	13	9.14e-14	ARHGAP27	3.46e-03	false	<0.05
				PLEKHM1	2.00e-02	false	<0.20
				KIAA1267	2.00e-02	true	<0.20
				ENSG00000214401	5.70e-01	false	>0.20
				NSF	6.40e-01	false	>0.20
				STH	7.30e-01	false	>0.20
				ARL17A	7.30e-01	false	>0.20
				ENSG00000204650	8.20e-01	false	>0.20
				WNT3	8.30e-01	true	>0.20
				CRHR1	8.50e-01	false	>0.20
				MAPT	8.60e-01	false	>0.20
				ENSG00000185294	9.30e-01	false	>0.20
				C17orf69	9.50e-01	false	>0.20
17:45518583-45874272	rs11651000	5	2.91e-12	TBX21	1.40e-01	true	>0.20
				C17orf57	2.40e-01	false	>0.20
				NPEPPS	3.30e-01	false	>0.20
				KPNB1	3.40e-01	false	>0.20
				TBKBP1	4.70e-01	false	>0.20
17:56398006-56410041	rs2526378	2	4.68e-09	MIR142	1.00e-02	false	<0.05
				BORCS8	3.30e-01	true	>0.20
17:79165171-79257880	rs2725391	4	1.31e-15	SLC38A10	3.00e-02	false	<0.20
				TEPSIN	2.90e-01	false	>0.20
				C17orf89	3.70e-01	false	>0.20
				CEP131	5.20e-01	true	>0.20
19:1614910-1658699	rs4807942	1	1.58e-08	TCF3	3.12e-03	true	<0.05
19:5822316-5845974	rs874232	4	8.12e-13	ENSG00000249707	7.00e-02	false	<0.20
				FUT3	4.50e-01	false	>0.20
				NRTN	6.60e-01	false	>0.20
				FUT6	8.40e-01	true	>0.20
19:19260586-19296217	rs7257072	1	1.98e-13	MEF2BNB	9.80e-01	false	>0.20
20:17818141-17833534	rs2745851	1	5.36e-13	MGME1	6.80e-01	true	>0.20
20:52170177-52212273	rs1555926	2	7.17e-09	ZNF217	4.43e-03	false	<0.05
				ENSG00000197670	8.60e-01	true	>0.20
21:36546756-36665202	rs7281587	1	1.21e-13	RUNX1	7.07e-03	false	<0.05
22:24093789-24182500	rs17630758	6	7.60e-41	VPREB3	4.11e-06	false	<=0.01
				DERL3	5.00e-02	false	<0.20
				CHCHD10	1.90e-01	false	>0.20

				MMP11	4.10e-01	false	>0.20
				SMARCB1	4.40e-01	true	>0.20
				C22orf15	9.40e-01	false	>0.20
22:39774448-39860868	rs5750830	3	3.43e-66	TAB1	6.70e-01	false	>0.20
				SYNGR1	9.80e-01	false	>0.20
				MGAT3	1.00e+00	true	>0.20

Appendix Table 10. SNPs associated with IgG glycosylation with nonsynonymous amino acid change.

Locus	gene	SNP	LD	p-value	Amino Acid Change	Ensembl Transcript	SIFT Descript	SIFT Score	PolyPhen Descript	PolyPhen Score
5:95211647-95347786	ELL2	rs3815768		1.537e-12	Ala => Thr	ENST00000513343<br>ENST00000237853	tolerated	0.66<br>1.00	benign	0.040<br>0.001
5:131026218-131833599	SLC22A4	rs1050152		6.099e-09	Leu => Phe	ENST00000200652	tolerated	0.14	benign	0.119
6:29299390-33883424	TCF19	rs7750641		5.859e-10	Pro => Ser	ENST00000376257<br>ENST00000376255<br>ENST00000542218	tolerated	0.34<br>0.34<br>0.55	benign	0.050<br>0.050<br>0.023
	TNXB	rs1150752		1.981e-09	Thr => Ala	ENST00000613214<br>ENST00000479795	tolerated	0.25, 0.28	benign	0.002<br>0.011
	HLA-DRA	rs7192		1.267e-09	Val => Leu	ENST00000374982<br>ENST00000395388	tolerated	0.21<br>0.26	benign	0.001<br>0.001
11:114323627-114450529	NXPE1	rs7944960	0.21	5.817e-11	Leu => Ser	ENST00000534921<br>ENST00000424269<br>ENST00000536312<br>ENST00000539878	tolerated	0.18<br>0.47<br>0.54<br>0.61	benign	0.013<br>0.013<br>0.036<br>0.013
	NXPE4	rs550897		1.057e-16	Tyr => His	ENST00000424261<br>ENST00000375478	tolerated	1.00<br>1.00	benign	0.000<br>0.000

17:37903731-38112190	ZPBP2	rs11557467	0.82	1.510e-14	Ser => Ile	ENST00000377940<br>ENST00000348931<br>ENST00000583811	tolerated	0.08<br>0.08<br>0.12	benign	0.004<br>0.173<br>0.142
	GSDMB	rs2305480	0.82	2.329e-14	Pro => Ser	ENST00000520542<br>ENST00000360317<br>ENST00000394179<br>ENST00000418519<br>ENST00000309481<br>ENST00000394175	tolerated	0.08<br>0.08<br>0.08<br>0.08<br>0.08<br>0.11	benign	0.356<br>0.242<br>0.242<br>0.242<br>0.242<br>0.110
		rs2305479		7.287e-14	Gly => Arg	ENST00000394179<br>ENST00000360317<br>ENST00000309481<br>ENST00000394175<br>ENST00000520542<br>ENST00000418519	deleterious	0.00<br>0.00<br>0.00<br>0.00<br>0.00<br>0.00	Probably damaging	0.996<br>0.995<br>0.996<br>0.993<br>0.997<br>0.995
17:43463492-44896083	CRHR1	rs16940665	0.91	2.937e-13	stop => Arg	ENST00000619154	NA,	NA	NA	NA
		rs16940674	0.80	8.507e-12	Ala => Val	ENST00000583888	Deleterious low confidence	0.00	unknown	0.000
	SPPL2C	rs12185233	1.00	1.397e-11	Arg => Pro	ENST00000329196	deleterious	0.01	Probably damaging	1.000
		rs12185268	1.00	6.428e-13	Ile => Val	ENST00000329196	tolerated	1.00	benign	0.001
		rs12373142	1.00	1.558e-11	Pro => Arg	ENST00000329196	Deleterious low confidence	0.01	benign	0.019
		rs12373142		1.558e-11	Pro => Leu	ENST00000329196	Tolerated low confidence	0.06	benign	0.004

	MAPT	rs754512<br>rs17651549<br><br><br><br><br>rs10445337	1.00<br>1.00	1.588e-11<br>1.610e-11<br><br><br><br><br>4.185e-13	Lys => stop<br>Arg => Trp<br><br><br><br><br>Ser => Pro	ENST00000576518<br>ENST00000415613<br>ENST00000262410<br>ENST00000571987<br>ENST00000344290<br>ENST00000262410<br>ENST00000571987<br>ENST00000344290<br>ENST00000415613	NA<br>deleterious<br><br><br><br>tolerated	NA<br>0.00<br>0.00<br>0.00<br>0.00<br>0.77<br>0.77<br>1.00<br>1.00	NA<br>Probably damaging<br><br><br>benign	NA<br>0.993<br>0.522<br>0.522<br>0.993<br>0.000<br>0.000<br>0.000<br>0.000
22:39774448-39860868	SYNGR1	rs4821888		2.171e-25	Thr => Ala	ENST00000415332	Tolerated low confidence	0.30	unknown	0.000

LD - linkage disequilibrium between the given and the SNP in the next row, as estimated with Broad Institute's SNAP tool. p-value - the lowest p-value for the given SNP in the discovery meta-analysis. In bold - replicated loci.

Appendix Table 11. Strongest eQTL for each probe in each cell type in the CEDAR dataset.

SNP	chromosome	Probe_Id	gene	tissue	P
rs8016322	14	ILMN_1741422	FUT8	CD14	4.78E-06
rs61085292	14	ILMN_1741422	FUT8	CD19	3.62E-04
rs1256519	14	ILMN_1741422	FUT8	CD4	7.02E-07
rs1256520	14	ILMN_1741422	FUT8	CD8	3.25E-06
rs6780016	3	ILMN_2384496	ST6GAL1	CD4	1.25E-03
rs13093508	3	ILMN_2384496	ST6GAL1	CD19	5.86E-04
rs4686837	3	ILMN_2384496	ST6GAL1	PLA	2.08E-08
rs55873928	3	ILMN_2384496	ST6GAL1	CD8	4.66E-03
rs35133002	3	ILMN_2384496	ST6GAL1	CD14	1.87E-05
rs56291008	3	ILMN_1756501	ST6GAL1	CD15	2.16E-04
rs13082825	3	ILMN_1756501	ST6GAL1	CD14	2.02E-03
rs4686837	3	ILMN_1756501	ST6GAL1	PLA	2.77E-25
rs4011973	3	ILMN_1756501	ST6GAL1	CD19	3.50E-04
rs28674898	3	ILMN_1756501	ST6GAL1	CD4	5.79E-06
rs58294975	3	ILMN_1756501	ST6GAL1	CD8	2.52E-05

SNP - variant with the strongest association with a given probe in a given cell type; gene - target gene of the given probe; P - association p-value of a given SNP and expression of the given probe.

Supplementary Figures

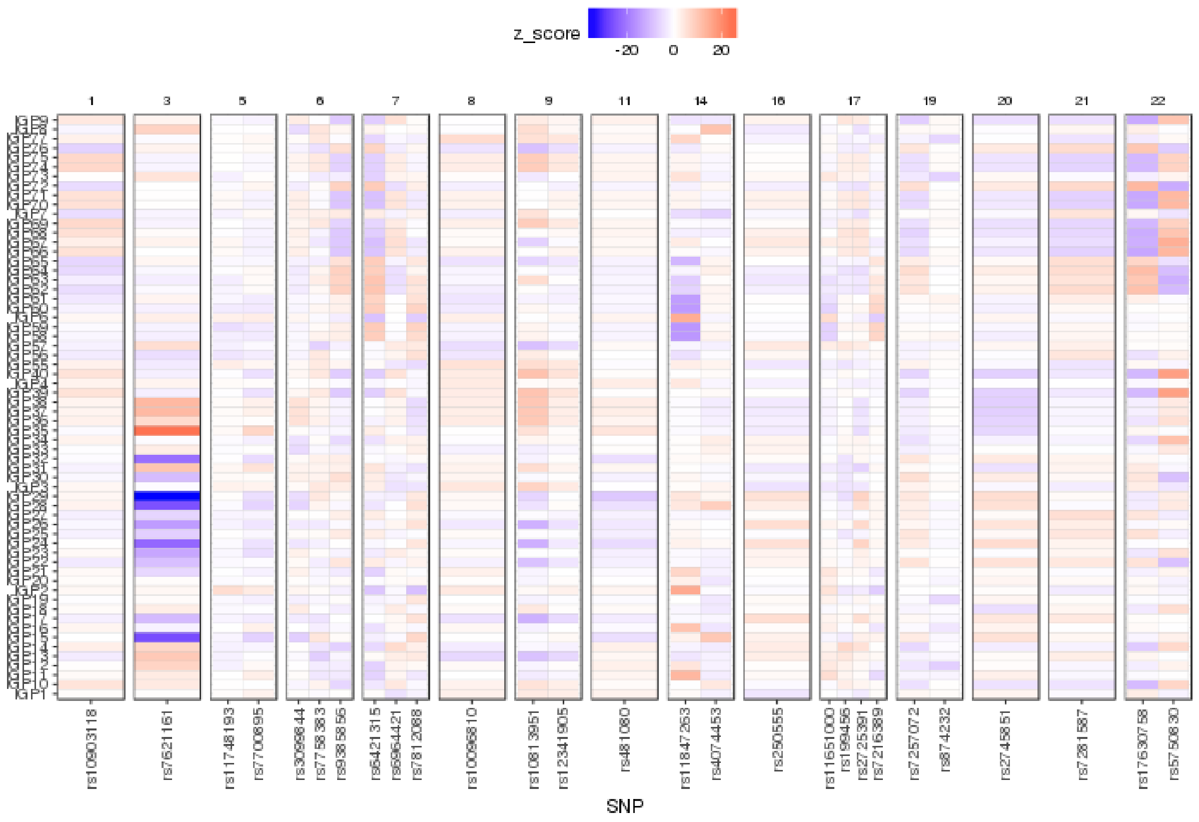

Fig. S1. Glycome-wide effect estimates of genome-wide significant loci. Each row represents z-score of effect estimate of a given glycan in all loci. Each column represents z-score of effect estimate of a given SNP in all glycans.

a)

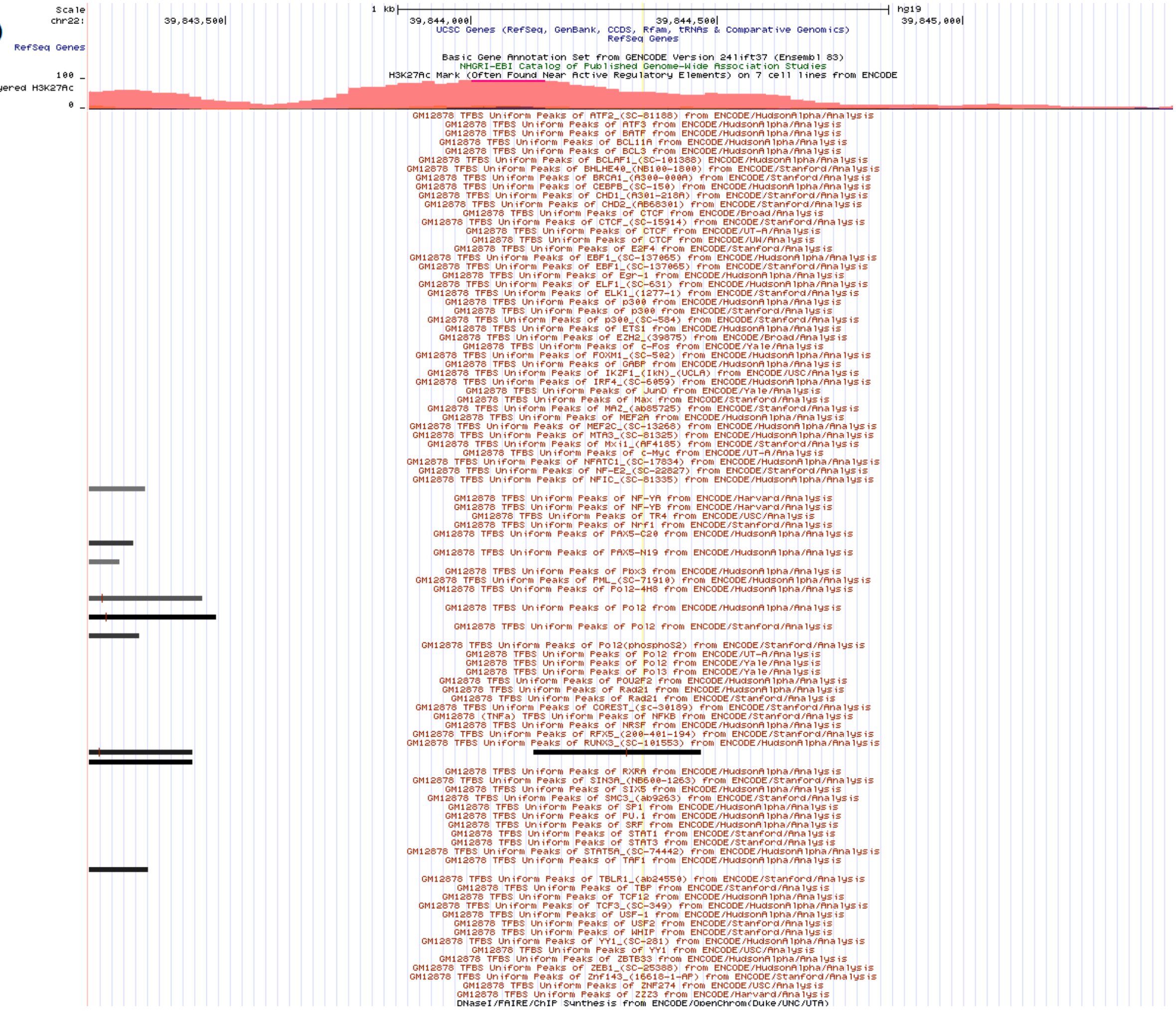

Fig. S2A. RUNX3 binding in the proximity of MGAT3 rs8137426, SNP strongly associated with IgG N-glycosylation is the region bound by RUNX3, in the proximity of MGAT3. Thick black lines represent transcription factor binding sites in the lymphoblastoid cell line GM12878 as observed in the ENCODE chip-data. Vertical yellow line represents position of the rs8137426. Layered H3K27AC panel shows that the area is likely to be transcriptionally active and marks a potential active enhancer. Data obtained from the UCSC genome browser in June 2017.

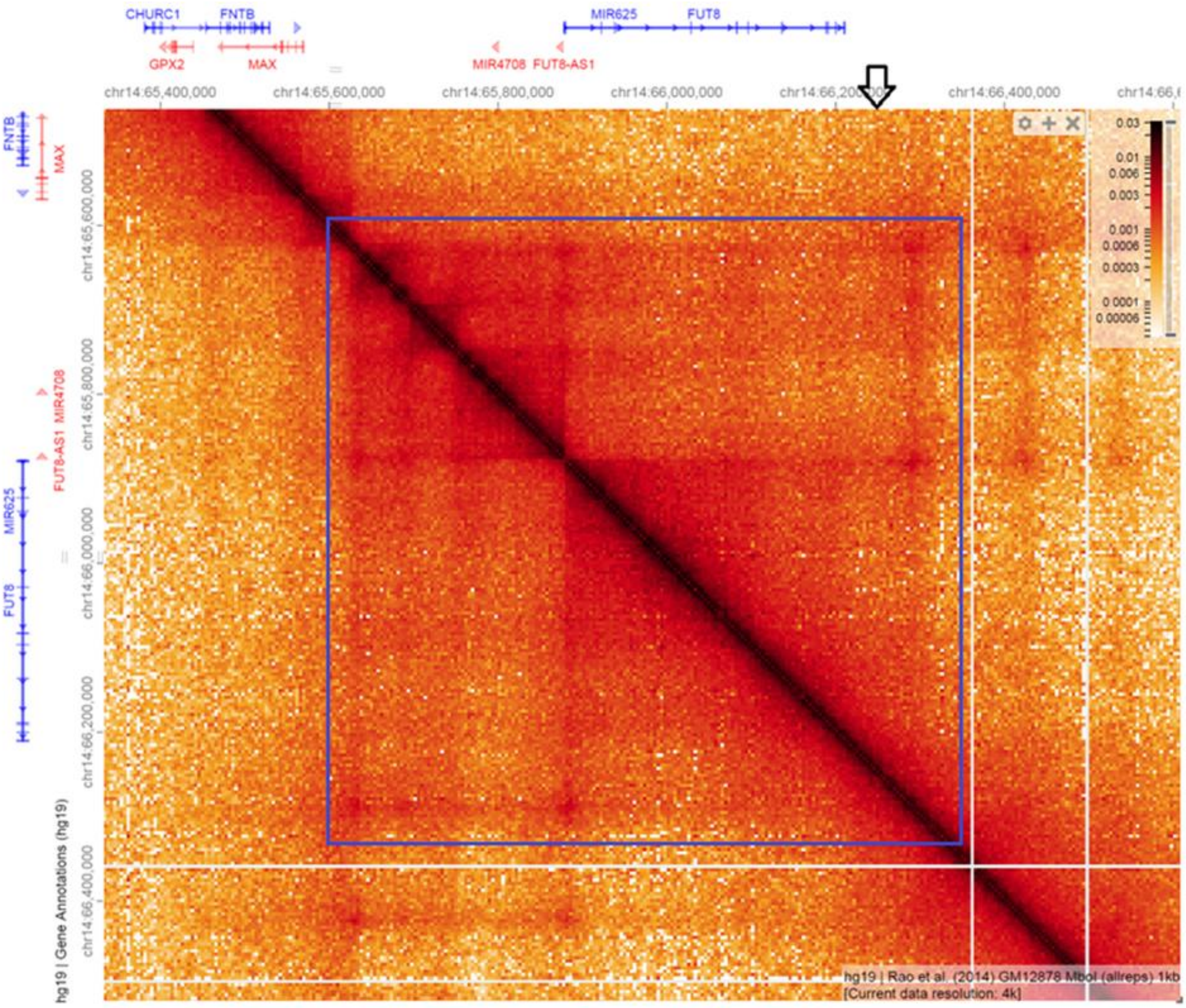

Fig. S2B. Top SNPs in *FUT8* locus lie in the same chromosomal topological associating domain as the transcription start site of FUT8. Hi-C profiling performed to 1 kb resolution in the LCL GM12878. Top *FUT8* SNPs are indicated by an arrow. Topological associating domain (TAD) is outlined in blue. Disruption of CTCF binding by SNPs in LD with rs4400971 may modify the interactions formed between enhancer and promoter regions of FUT8. Figure obtained from http://higlass.io/app/[134].
